# Hypotaurine Reduces Glucose‐Mediated Vascular Calcification

**DOI:** 10.1111/apha.70075

**Published:** 2025-07-07

**Authors:** Marina A. Heuschkel, Armand Jaminon, Steffen Gräber, Anna Artati, Jerzy Adamski, Joachim Jankowski, Leon Schurgers, Nikolaus Marx, Willi Jahnen‐Dechent, Claudia Goettsch

**Affiliations:** ^1^ Department of Internal Medicine I – Cardiology Medical Faculty, RWTH Aachen University Aachen Germany; ^2^ Department of Biochemistry Cardiovascular Research Institute Maastricht (CARIM), Maastricht University Maastricht the Netherlands; ^3^ Helmholtz Institute for Biomedical Engineering, Biointerface Lab Aachen Germany; ^4^ Metabolomics and Proteomics Core Helmholtz Center Munich, German Research Center for Environmental Health Neuherberg Germany; ^5^ Institute of Experimental Genetics, Helmholtz Center Munich, German Research Center for Environmental Health Neuherberg Germany; ^6^ Department of Biochemistry Yong Loo Lin School of Medicine, National University of Singapore, Singapore, Singapore Institute of Biochemistry, Faculty of Medicine, University of Ljubljana, Ljubjana Slovenia; ^7^ Institute for Molecular Cardiovascular Research IMCAR, RWTH Aachen University Aachen Germany; ^8^ Institute of Physiology, Medical Faculty Carl Gustav Carus, Technical University Dresden Dresden Germany

**Keywords:** hypotaurine, metabolomics, transcriptomics, vascular calcification

## Abstract

**Aim:**

Vascular calcification (VC), a characteristic feature of peripheral artery disease in patients with diabetes and chronic kidney disease, has been associated with poor prognosis. We hypothesize that hyperglycemia drives VC through alterations in metabolomic and transcriptomic profiles.

**Methods:**

Human coronary artery smooth muscle cells (SMCs) were cultured with 0, 5.5, and 25 mM glucose under calcifying conditions. Untargeted metabolomic and transcriptomic analyses were performed at different time points. Mitochondrial respiration was examined using Seahorse analysis.

**Results:**

Glucose‐treated SMCs promoted extracellular matrix (ECM) calcification in a concentration‐ and time‐dependent manner. The absence of glucose entirely abolished SMC calcification but reduced SMC proliferation in control and calcifying conditions compared to 25 mM glucose. Multi‐omics data integration revealed key players from the hypotaurine/taurine metabolic pathway as the center hub of the reconstructed network. Glucose promoted the hypotaurine secretion, while its intracellular abundance was not altered. Blocking hypotaurine production by propargylglycine increased ECM calcification, while hypotaurine treatment prevented it. Furthermore, omics data suggest energy remodeling in calcifying SMCs under hyperglycemia. Calcifying SMCs exhibited decreased oxygen consumption that was partially restored by hypotaurine. Validation of our in vitro models using the murine warfarin model demonstrated reduced hypotaurine/taurine transporter (TAUT) expression in SMCs.

**Conclusions:**

Our multi‐omics analysis revealed a role of the hypotaurine/taurine metabolic pathway in glucose‐induced SMC calcification. Moreover, our data suggest a glucose‐dependent energy remodeling in calcifying SMCs and that increasing glucose concentrations fuel ECM calcification. Our work highlights potential novel therapeutic targets that warrant further investigation in hyperglycemia‐dependent in vitro SMC calcification.

## 1 Introduction

Type 2 diabetes mellitus (T2DM) is a metabolic disorder characterized by insulin resistance, with dysfunction of pancreatic β‐cells due to unsettled hyperglycemia [1]. It is estimated that globally, 1 in 11 adults aged 20–79 years has diabetes mellitus [2]. This incidence is projected to rise from 415 million in 2015 to 642 million by 2040 [2]. The significant comorbidity in T2DM patients is cardiovascular disease (CVD) [3]. Medial arterial calcification is a strong independent predictor of total cardiovascular mortality, particularly in individuals with T2DM [4, 5].

There is currently no pharmacological treatment for vascular calcification (VC), a process in which mineral deposits in the tunica intima or tunica media of arterial blood vessels cause tissue stiffening, plaque vulnerability, and heart failure [6]. Clinically, there is a link between T2DM and VC [7]. Patients with T2DM have a significantly increased prevalence of VC compared to non‐diabetic controls [8]. Moreover, there is an established connection between hyperglycemia, aortic calcium score, and diagnostic glycated HbA1c as predictors of CVD [9, 10]. Nonetheless, a detailed understanding of the underlying molecular mechanism and the role of metabolic control of T2DM‐induced VC is limited [7].

The predominant cell type in the artery wall is the vascular smooth muscle cell (SMC). An increasing body of data suggests that SMCs have remarkable plasticity. Their ability to switch between different phenotypes is crucial in vascular (dys)function, including VC [11]. In the apparently healthy vessel, SMCs are contractile and characterized by low proliferation rates, quiescence, and high expression of key contractile proteins [12]. However, under specific calcification stimuli, SMCs can differentiate into a synthetic osteoblast‐like phenotype characterized by decreased contractile marker expression, increased proliferation, and migration [13]. Various mechanisms, encompassing oxidative stress and inflammation, contribute to the transdifferentiation of SMCs to an osteoblast‐like phenotype [14, 15].

Metabolic reprogramming is a central hallmark of CVD, as is the transdifferentiation of SMCs during calcification, particularly in the context of hyperglycemia, which drives changes in glucose and lipid metabolism [16]. These metabolic shifts lead to increased production of reactive oxygen species (ROS), contributing to oxidative stress and inflammation, both promoting the osteogenic transdifferentiation of SMCs and subsequent calcification of the vascular wall [15, 17]. A better understanding of these metabolic adaptations can reveal new therapeutic targets for preventing or mitigating VC in T2DM patients. As a result, the reconstruction and profiling of metabolic pathways are a promising approach to understanding the underlying mechanisms of VC. The present study investigated the metabolic changes in SMCs driving glucose‐dependent calcification through an untargeted multi‐omics approach. By combining multiple omics levels, we dissected the molecular basis of glucose‐dependent in vitro SMC calcification from mRNA transcription (transcriptome) to metabolic regulation (intracellular metabolome) and metabolite secretion (extracellular metabolome).

## 2 Materials and Methods

### 2.1 Animals

DBA/2 mice were bought from Charles River Laboratories Inc. (‘s‐Hertogenbosch, The Netherlands). Mice were kept in a temperature‐controlled environment (20°C) with a regular day and night cycle. The animal protocol was approved by the ethics committee for animal experiments of Maastricht University.

The warfarin diet was prepared as described previously [18]. In brief, warfarin (3 mg/g; Sigma) supplemented with vitamin K1 (1.5 mg/g; Sigma) was mixed with standard chow diet (Arie Blok diets). Experimental animals (10‐to 12‐weeks‐old males) were weighed, and mice were placed on a warfarin diet for either 0 weeks (control, *n* = 6) or 4 weeks (warfarin group, *n* = 6). At sacrifice, the aorta was flushed with 100 μM sodium nitroprusside in phosphate‐buffered saline (PBS). The aortic arch was isolated, fixed in 1% paraformaldehyde (PFA) overnight, and embedded in paraffin.

### 2.2 Human Vascular Smooth Muscle Cell Culture

Human primary coronary artery smooth muscle cells (pSMC, Promocell) were cultured in Smooth Muscle Cell Growth Medium 2 (Promocell) and supplemented with Smooth Muscle Cell Growth Medium 2 Supplement Mix (Promocell), consisting of epidermal growth factor (0.5 ng/mL), insulin (5 μg/mL), basic fibroblast growth factor‐B (2 ng/mL), 5% fetal bovine serum (FBS), and 1% penicillin–streptomycin (P/S).

As previously described, immortalized vascular smooth muscle cells (imSMC) were generated from human primary coronary artery SMC [19]. imSMC were cultured in growth media (Dulbecco's Modified Eagle Medium (DMEM, 4.5 g/L glucose, 0.58 g/L L‐glutamine, without pyruvate) with 10% FBS and 1% P/S).

Human ventral smooth muscle cells were isolated from non‐atherosclerotic abdominal aortas of surgical biopsies in accordance with Maastricht University Medical Centre research and diagnostic procedure, as previously described. Collection, storage, and use of tissue and patient data were performed in agreement with the Dutch Code for Proper Secondary Use of Human Tissue [20].

Cells were maintained at 37°C in humidified 5% CO_2_ until 80% confluence. pSMCs were used up to passage ten and imSMCs up to passage 30. For the experiments, cells were cultured in DMEM containing no glucose (CM; ThermoFisher, cat. 11 966 025), no glutamine, and no sodium pyruvate with 10% FBS and 1% P/S. Glucose (Sigma‐Aldrich), D‐mannitol (Sigma‐Aldrich), hypotaurine (Sigma‐Aldrich), and DL‐propargylglycine (PAG, Sigma‐Aldrich) were added to the media as indicated.

### 2.3 RNA Interference

Gene silencing was achieved by transfecting imSMC with 50 nM siRNA against SLC6A6 (ON‐TARGETplus Human SLC6A6 siRNA—SMARTpool, Horizon Discovery) or non‐targeting siRNA (ON‐TARGETplus Non‐Targeting Pool, Horizon Discovery) using DharmaFECT 1 (Dharmacon) in Opti‐MEM medium (Thermo Fisher Scientific). Transfection was performed twice per week over the entire cell culture period.

### 2.4 In Vitro Calcification Assay and Visualization

For induction of calcification, cells were cultured in calcium/phosphate‐enriched media (CaP), which consisted of CM supplemented with 1.8 mM CaCl_2_ (ROTH) and 0.9 mM NaH_2_PO_4_. H_2_O (ROTH) to obtain a final concentration of 3 mM calcium and 2 mM phosphate. Glucose and mannitol were added as described in the experiments. The media was changed every 3 days. The mineralized extracellular matrix (ECM) was assessed by Alizarin Red S staining. Cells were fixed with 4% PFA and stained with 2% Alizarin Red S (pH 4.2, Sigma‐Aldrich) for 30 min at room temperature. Excess dye was removed by washing with distilled water and imaged under a light microscope (EVOS FL Cell Imaging System). For the quantification of the Alizarin red‐stained matrix, 100 mM cetylpyridinium chloride (Sigma‐Aldrich) in 10 mM sodium phosphate buffer (pH 7.0) was added to each well and incubated for 30 min at 37°C. The eluted stain was measured at 570 nm using a spectrophotometer (TECAN; Infinite M200, i‐control software).

Additionally, real‐time ECM calcification was monitored using a live‐cell fluorescence imaging system with a Fetuin‐A–Alexa Fluor 546 probe. This probe, which binds to mineral deposits, was used to track the progression of calcification over time. Fetuin‐A–Alexa Fluor 546 (final concentration: 1 μg/mL) and Hoechst 33,342 (1 μg/mL; Invitrogen) were added to the culture medium at the start of the experiment, following previously established protocols [20]. Imaging was performed on day 7 using the Cytation 3 system (BioSPX) and Gen5 software version 2.9 (BioTek).

For alizarin red staining on paraffin‐embedded murine aortic arches, the tissues were sectioned, rehydrated, and incubated in 2% alizarin red S solution for 2 min. Images were captured using an EVOS m5000 microscope (Invitrogen) at 4‐ and 20‐times magnification.

### 2.5 Calcium Measurement

For the experiment without cells, CM or CaP media with different glucose and mannitol concentrations were maintained in culture dishes (37°C in humidified 5% CO_2_) for 7 days. The media was changed every three days to reproduce the standard experimental operations. Subsequently, the supernatant was collected and centrifuged at 20,000 g for 20 min at room temperature. Then, the supernatant was discarded, and the pellet was resuspended in 0.1 N HCl overnight (37°C in humidified 5% CO_2_) for calcium elution. The calcium content in the supernatant was determined calorimetrically using a calcium colorimetric assay kit (BioVision), according to the manufacturer's instructions.

### 2.6 Cell Viability

Cell viability was assessed with a live/dead fluorescence‐based cell assay using fluorescein diacetate (FDA)/propidium iodide (PI). Cells were incubated with FDA (50 μg/mL, Sigma‐Aldrich) and PI (50 μg/mL, Sigma‐Aldrich) in PBS for 30 s at 37°C in humidified 5% CO_2_. Live images were acquired using the FITC filter for FDA (excitation/emission, 535/585 nm) and rhodamine for PI (excitation/emission, 605/660 nm) with a Leica DMI6000 fluorescence microscope. Stimulation of cells with 0.05% Triton X‐100 (Sigma‐Aldrich) for 2 min was used as a positive control for dead cells.

Furthermore, cell viability was assessed using the resazurin‐based Alamar Blue assay (Invitrogen), according to the manufacturer's instructions. The fluorescence was measured at 560 nm_Ex_/590 nm_Em_ (TECAN).

The ApoTox‐Glo Assay (Promega) was used to determine apoptosis, following the manufacturer's instructions. A caspase‐3/7 substrate was added, producing a luminescent signal proportional to the caspase activity. Staurosporine (Sigma‐Aldrich; 0.2 μM, 24 h) was used as a positive control for the apoptosis assay. Luminescence was measured with a microplate reader (TECAN).

### 2.7 Cell Proliferation Assay

Cell proliferation was determined using the 5‐bromo‐2‐deoxyuridine (BrdU) assay. The protocol followed the manufacturer's instructions (Sigma‐Aldrich). Cells were labeled with 100 μM BrdU for 16 h at 37°C in humidified 5% CO_2_. The reaction product was quantified by measuring the absorbance at 370 nm (TECAN).

### 2.8 Hydrogen Peroxide Measurement

The Amplex Red Hydrogen Peroxide/Peroxidase Assay Kit (Invitrogen) was used for H_2_O_2_ measurement according to the manufacturer's instructions. Fluorescence was measured with excitation/emission set at 530 nm/560 nm (TECAN). The amount of H_2_O_2_ was normalized to protein content assessed by BCA assay.

### 2.9 Live Mitochondria Fluorescence Labeling

Mitochondria of live cells were labeled with 300 nM MitoTracker Red FM (Thermo Fisher Scientific) in CM for 30 min (37°C in humidified 5% CO_2_). Nuclear staining was performed with 1 μM Hoechst 33342 solution in CM for 15 min (37°C in humidified 5% CO_2_). Images were acquired using a Leica DMi6000B inverted fluorescence microscope in the absorption/emission spectra of 581/644 nm and 350/451 nm, respectively.

### 2.10 RNA Preparation and Real‐Time PCR

Total RNA was isolated using TRIzol (Life Technologies). Reverse transcription was performed using the High‐Capacity cDNA Reverse Transcription Kit (Life Technologies), according to the manufacturer's protocol. The gene expression levels were quantified by TaqMan‐based real‐time PCR reactions (Life Technologies). The used TaqMan probes are listed in Table S1. The expression levels were normalized to RPLP0. Results were calculated using the ΔCt method and presented as fold change relative to control.

### 2.11 Western Blot

Cells were lysed using RIPA buffer (Thermo Scientific) supplemented with protease and phosphatase inhibitors (Roche). Protein levels were quantified with the BCA assay (Thermo Scientific), following the manufacturer's guidelines. A total of 15 μg of protein per sample was loaded onto an 8% polyacrylamide gel for electrophoresis, followed by transfer to a nitrocellulose membrane. Membranes were incubated overnight with primary antibodies targeting (hypo)taurine sodium and chloride transporters SLC6A6 (protein: TAUT) (1:100; Santa Cruz, sc‐393 036) and beta‐actin (1:10000; Sigma‐Aldrich, A2228) as a loading control. Detection was carried out using an HRP‐linked secondary antibody (anti‐mouse, Cell Signaling, #7076), and signal was developed using the SuperSignal West chemiluminescent substrate (ThermoFisher Scientific). Bands were visualized with a ChemiDoc MP Imaging System and Image Lab software (version 6.0). Densitometric analysis was performed using FIJI (ImageJ, version 1.53c), with protein levels normalized to beta‐actin.

### 2.12 Immunohistochemistry

Paraffin‐embedded murine aortic arches were sectioned and stained for TAUT. After rehydration, antigen retrieval was performed by heating samples in 10 mM sodium citrate (pH 6.0 set with saturated citric acid). Subsequently, tissues were blocked in 5% goat serum and incubated at 4°C with primary TAUT antibody overnight (1:200; Proteintech). Subsequently, sections were incubated for 1 h with HRP‐conjugated secondary antibody (Brightvision+ anti‐IgG poly HRP; Immunologic), detected with NovaRED for 2 min (Vector Labs), and counterstained with hematoxylin (Klinipath). Images were captured using an EVOS m5000 microscope (Invitrogen) at 4‐ and 20‐times magnification.

Image J v2.0 software was employed to quantify the mean intensity of TAUT staining. TAUT raw intensity was normalized to tissue area.

### 2.13 Transcriptomics

Since the osmotic control showed no significant difference compared to the glucose treatment regarding its calcification profile, we proceeded with the transcriptomics analysis considering only the glucose conditions. Three independent pSMC donors were cultured in control media (CM; 5.5 mM glucose) or CaP with 0 mM, 5.5 mM, and 25 mM glucose for 3 days. Three hundred ng of total RNA was processed using the GeneChip WT PLUS Reagent Kit (Affymetrix Inc.) following the manufacturer's protocol to yield purified biotinylated sense‐stranded cDNA. Hybridization was performed on Clariom D Human Arrays using the GeneChip Hybridization, Wash & Stain Kit (Affymetrix Inc.) and Fluidics Station 450 for 16 h at 45°C. Arrays were scanned using Affymetrix GeneChip Scanner 3000 controlled by GeneChip Command Console version 4.0 to produce CEL intensity files. The raw data were analyzed using the Transcriptome Analysis Console software (TAC4.0, ThermoFisher Scientific) with default parameters for gene‐level expression analysis based on the annotation Hg38 clariom_D_Human.r1.na36.hg38.a1.transcript.csv. SST‐RMA was applied for normalization and summarization. Values are defined as log2‐scaled normalized gene‐level expression values. Genes were considered differentially expressed when the fold change was ±1.2, *p* < 0.05 based on the empirical Bayes statistics (eBayes) function from the limma R package. Microarray data can be accessed using GEO Series accession number GSE283693.

### 2.14 Metabolomics

As described for the transcriptomics, pSMC from the same three independent cell donors were seeded at 1,40,000 cells/mL density, and cells were treated with the corresponding glucose and calcification condition for 3 or 5 days. The supernatant was collected and centrifuged at 20,000 g for 20 min and stored at −80°C until metabolomics analysis.

The cells were quickly washed twice with 2 mL warm PBS, and their metabolism was subsequently quenched by the addition of 400 μL pre‐cooled (dry ice) extraction solvent, an 80/20 (v/v) methanol/water mixture that contained four standard compounds (final concentration: 0.25 mg/mL tridecanoic acid, 2.5 μg/mL DL‐2‐fluorophenylglycine, 0.25 mg/mL d6‐cholesterol, and 100 μg DL‐4‐chlorophenylalanine) for monitoring the efficiency of metabolite extraction. Cells were scraped off the culture vessel using rubber‐tipped cell scrapers and collected with the solvent into pre‐cooled microtubes. The culture well was rinsed with another 100 μL extraction solvent, and the liquid was also transferred to the tube. The samples were stored at −80°C until metabolomics analysis.

Metabolomics were performed at the metabolomics core facility, Helmholtz Zentrum Munich, Germany. 160 mg glass beads (0.5 mm, VK‐05, Peqlab) were added to the cell samples in the microtubes (2.0 mL, Sarstedt). Cells in the tubes were then homogenized using the Precellys24 homogenizer at 0°C–4°C twice over 25 s at 5500 rpm with a 5 s pause interval. Briefly, 20 μL of the sample was taken for the DNA content determination. Subsequently, the remaining cell samples in the tubes were centrifuged at 4°C and 11,000 x g for 5 min, and 100 μL of the supernatant was aliquoted onto four 96‐well microplates. Two aliquots were used for two separate reverse phase (RP)/UPLC‐MS/MS methods with positive ion mode electrospray ionization (ESI), 1 was used for analysis by (RP)/UPLC‐MS/MS with negative ion mode ESI. The supernatant aliquots were dried on a TurboVap 96 (Zymark, Sotax, Lörrach, Germany).

For cell medium: 100 μL of medium was pipetted into a 2 mL 96‐well plate. Protein was precipitated, and the metabolites in the supernatant samples were extracted with 500 μL methanol, containing the four recovery standards to monitor the extraction efficiency. After centrifugation, the supernatant was split into four aliquots of 100 μL each onto two 96‐well microplates. Two for analysis by two separate reverse phases (RP)/UPLC‐MS/MS methods with positive ion mode electrospray ionization (ESI), and one for analysis by (RP)/UPLC‐MS/MS with negative ion mode ESI. Sample extracts were dried on a TurboVap 96 (Zymark, Sotax, Lörrach, Germany). Liquid handling was performed on an automated MicroLab STAR robot (Hamilton Bonaduz AG, Bonaduz, Switzerland) to minimize human error.

Before the UPLC‐MS/MS analysis, the dried samples were reconstituted in acidic or basic LC‐compatible solvents, each containing eight or more standard compounds at fixed concentrations to ensure injection and chromatographic consistency.

The UPLC‐MS/MS platform utilized a Waters Acquity UPLC with Waters UPLC BEH C18‐2.1 × 100 mm, 1.7 μm columns; a Thermo Scientific Q Exactive high‐resolution/accurate mass spectrometer interfaced with a heated electrospray ionization (HESI‐II) source; and an *N* Orbitrap mass analyzer operated at 35,000 mass resolution. One aliquot of the extracts was reconstituted in acidic positive ion conditions, chromatographically optimized for more hydrophilic compounds (for early eluting compounds). In this method, the extracts were gradient eluted from the C18 column using water and methanol containing 0.05% perfluoropentanoic acid (PFPA) and 0.1% formic acid (FA). Another aliquot that was also analyzed using acidic positive ion conditions but was chromatographically optimized for more hydrophobic compounds (for later eluting compounds) was gradient eluted from the same C18 column using methanol, acetonitrile, and water containing 0.05% PFPA and 0.01% FA and was operated at an overall higher organic content. The basic negative ion condition extracts were gradient eluted from a separate C18 column using water and methanol containing 6.5 mM ammonium bicarbonate at pH 8.

The MS analysis alternated between MS and data‐dependent MS2 scans using dynamic exclusion and a scan range of 80–1000 *m/z*. Metabolites were identified by automated comparison of the ion features in the experimental samples to a reference library of chemical standard entries that included retention time, molecular weight (*m/z*), preferred adducts, and in‐source fragments, as well as associated MS spectra, and curation by visual inspection for quality control using proprietary software developed by Metabolon Inc.

We normalized the metabolomics data from cell homogenates for differences in cell number by assessing the DNA content using a fluorescence‐based assay for DNA quantification as previously described [21].

### 2.15 Bioinformatics Analysis

For transcriptomics data, gene symbol was used as the identifier type. The intersection of different gene datasets (fold change ±1.2, *p* < 0.05) was obtained through Venn diagrams (https://bioinformatics.psb.ugent.be/webtools/Venn/).

Overrepresentation analysis (genes with fold change ±1.5, *p* < 0.05) was performed using the web‐based tool ConsensusPathDB database (http://consensuspathdb.org), employing the canonical pathways from the KEGG and Reactome databases [22]. Pathways with FDR value < 0.001 were considered significantly enriched in a gene set of interest containing at least three enriched genes.

Metabolomics data were analyzed using the web‐based tool MetaboAnalyst 4.0 (https://www.metaboanalyst.ca; date: 11/2024) [23]. For fold change analysis between two groups, data was uploaded as concentrations and “compound name” nomenclature; no filtering or sample normalization was applied in the software. Principal component analysis (PCA) was performed and displayed as a 2D score plot and biplot. Next, fold change was calculated as the ratios between two group means with a threshold of ±1.2. An unpaired *t*‐test with equal group variance and a raw *p* threshold of < 0.05 was applied for statistical analysis. Pathway enrichment analysis was performed using the KEGG pathway library in metabolite sets containing at least two entry metabolites using the hypergeometric test. Pathways with *p* < 0.001 were considered to be significantly enriched.

Multi‐omics data integration was performed using the web‐based tool OmicsNet (https://www.omicsnet.ca) [24]. Genes were uploaded based on corresponding official gene symbols and metabolites based on KEGG nomenclature. Only connector genes that targeted seeded nodes were considered using the IntAct interaction database for manually curated, experimentally validated PPI. The multi‐sourced integrated database ‘Relational database of Metabolic Pathways’ was assessed for overrepresentation analysis, and pathways with FDR value < 0.001 were considered significantly enriched.

### 2.16 Real‐Time Extracellular Efflux Analysis

The bioenergetic profile of cells was assessed through extracellular efflux analysis using the Seahorse XFe96 Flux Analyzer (Agilent) with the Seahorse XF Cell Mito Stress Test Kit (Agilent). This technique allows real‐time measurements of the oxygen consumption rate (OCR) in living cells. imSMCs were seeded into XF96 cell culture microplates (Agilent) at ∼5000 cells per well and cultured for 7 days in the corresponding treatments (37°C, 5% CO_2_). One day before the assay, the XFe96 Sensor Cartridge (Agilent) was hydrated with water overnight at 37C in a CO_2_‐free incubator. Cells were washed with DMEM supplemented with 10 mM glucose, 2 mM L‐glutamine, and 1 mM pyruvate, pH 7.4, and incubated in a CO_2_‐free incubator at 37C for one hour. The Sensor Cartridge was loaded with different inhibitors from the Seahorse Mito Stress Kit: oligomycin (final concentration: 1 μM), carbonyl cyanide‐4 (trifluoromethoxy) phenylhydrazone (FCCP; final concentration: 0.5 μM), and rotenone/antimycin A (final concentration: 0.5 μM). The protocol of the measurements includes three baseline measurements with mix (three minutes)/measure (three minutes), followed by the injection of port A (oligomycin). Afterward, respiration was measured three times with mix (three minutes)/measure (three minutes) with the injection of ports B (FCCP) and C (rotenone/antimycin) with the same measurement cycle of three times mix/measure. OCR measurements were normalized to protein content assessed by the BCA assay.

### 2.17 Nephelometer Assay

The nephelometer assay was performed as previously described [25]. Stock solutions were preheated for 1 h in a 37°C water bath, and 1 h prior to start, the stock solutions were equilibrated to 34°C in a temperature‐controlled room. All following pipetting steps were performed with a Liquidator96 bench‐top pipetting system. Pipetting was performed in the following order: (1) NaCl/PO4 solution (8 mL of 140 mM NaCl solution was mixed with 20 mL phosphate solution (19.44 mM Na_2_HPO_4_ + 4.56 mM Na_2_HPO_4_ + 100 mM Hepes + 140 mM NaCl pH‐adjusted with 10 M NaOH to 7.40 at 37°C)): 70 μL/well, (2) shaking for 1 min on a plate shaker at 1200 rpm, (3) calcium solution (40 mM CaCl_2_ + 100 mM Hepes+140 mM NaCl pH‐adjusted with 10 M NaOH to 7.40 at 37°C): 50 μL/well, (4) shaking for 1 min at 1200 rpm. The measurements were performed using a Nephelostar instrument (BMG, Germany) controlled via the Galaxy software. The assay was conducted at 36.5°C–37°C over 200 cycles, with each cycle consisting of a 1.5 s measurement time per well and a position delay of 0.1 s. The instrument operated in horizontal plate reading mode, with a total cycle time of 180 s per cycle.

### 2.18 Statistical Analysis

Data are presented as mean ± SD; *n* indicates the number of independent experiments. Statistical analyses were performed using the GraphPad Prism program (Prism Software Inc., Version 8). For comparison between two groups, an unpaired Student's *t*‐test with equal variance was performed. For comparison among three or more treatment groups, one‐way ANOVA followed by Dunnett or Sidak post‐test was performed. In case of unequal variance detected by the Brown–Forsythe *F*‐test, unpaired Welch's correction was used. A *p* value of less than 0.05 was considered significant.

## 3 Results

### 3.1 Glucose Promotes Vascular Smooth Muscle Cell‐Extracellular Matrix (ECM) Calcification Concentration‐Dependently

**FIGURE 1:**
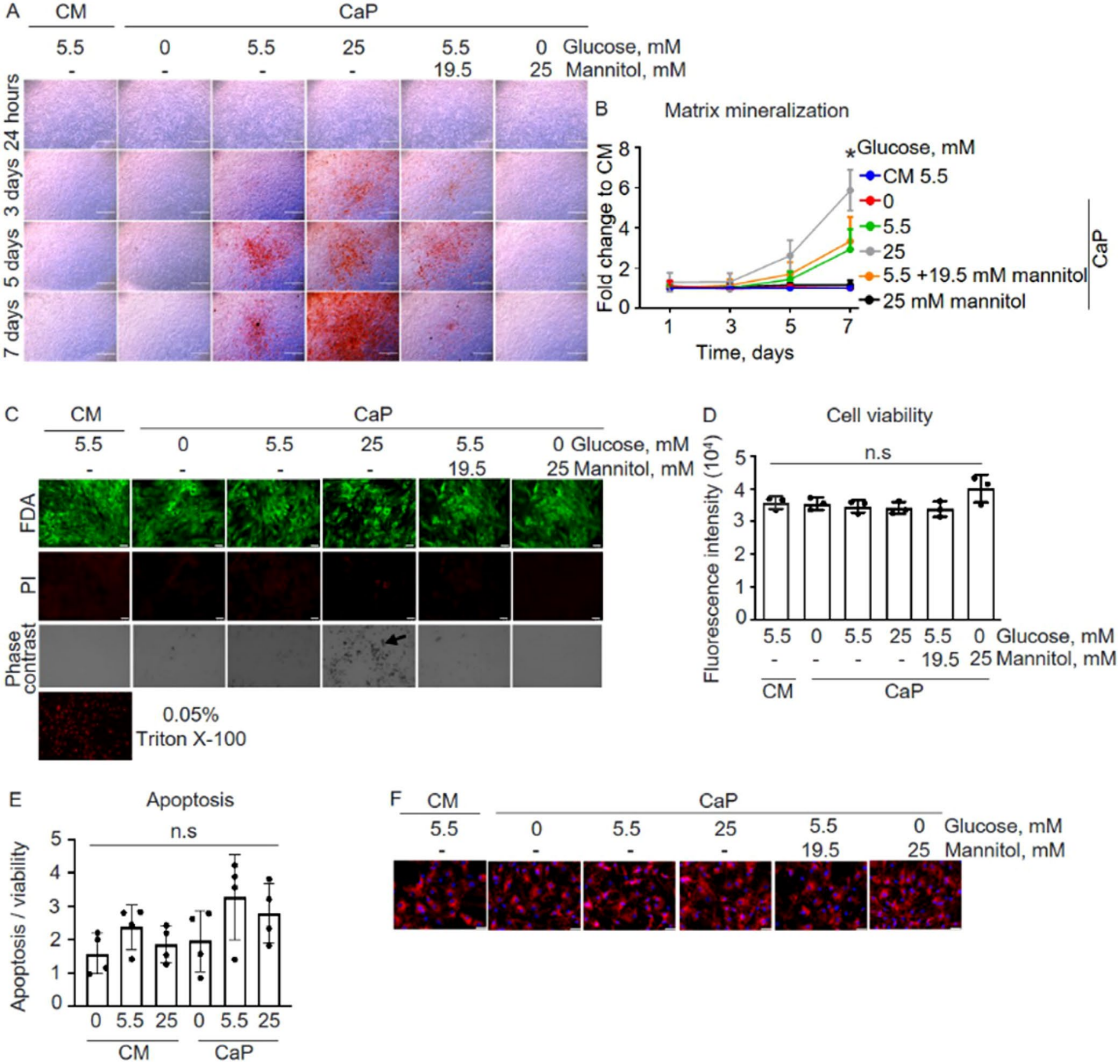
Glucose promoted extracellular matrix (ECM) calcification in primary human coronary artery smooth muscle cells (pSMC) concentration‐ and time‐dependently. pSMC were cultured in control (CM; 5.5 mM glucose) or calcium/phosphate (CaP)‐enriched media with 0, 5.5, or 25 mM glucose over 7 days. Mannitol served as osmotic control. (A) Representative images of ECM mineral accessed by Alizarin Red staining after treatment with glucose and mannitol in CM or CaP media for 1, 3, 5, and 7 days. Scale bars: 1000 μm. (B) Quantification of eluted Alizarin Red staining from the ECM from Figure 1A. Two‐way ANOVA with Dunnett's post hoc test. *p < 0.05 compared to all other conditions at day 7. (C) Representative images of cell viability visualized with fluorescein diacetate (FDA) and propidium iodide (PI) dual immunofluorescence staining at day 7. Negative control: Permeabilized cells with 0.05% Triton X‐100. Phase contrast shows the mineral indicated by the arrow. Scale bars: 75 μm. (D) Cell viability assessed by AlamarBlue assay. pSMC were cultured with glucose and CaP media for 7 days. (E) Apoptosis at day 7. (F) Representative images of the mitochondria‐specific dye MitoTracker Red (red) and nuclear Hoechst staining (blue) at day 7. Scale bars: 75 μm. *n* = 3–4 in duplicates, each *n* represents an independent pSMC donor. Mean ± SD. One‐way ANOVA with Dunnett's post hoc test compared to 5.5 mM glucose control (CM), n.s.; not significant.

An initial investigation was performed to determine whether glucose promotes SMC calcification. To this end, calcifying SMCs were treated with increasing glucose concentrations (0, 5.5, 25 mM) for up to 7 days. Glucose promoted ECM calcification in a time‐ and concentration‐dependent manner (Figure 1A,B). On day 3, in the presence of 25 mM glucose, SMCs exhibited the first detectable matrix calcification (*p* < 0.001), which further increased to 2.5‐fold and 5.8‐fold (*p* < 0.001) on days 5 and 7, respectively, compared to SMCs cultured without glucose (0 mM). Notably, the absence of added glucose in the culture medium prevented matrix calcification. Of note, when osmotic controls using mannitol were used, similar effects were observed at corresponding glucose concentrations, suggesting that the observed phenotype is not due to an osmotic effect. Cell viability and apoptosis of SMCs were unaffected by CaP or varying glucose concentrations (Figure 1C–E).

Since hyperglycemia is known to disrupt the morphology of mitochondria in various cell types, including SMCs [26, 27], we evaluated the mitochondrial phenotype of calcifying SMCs. However, no differences in mitochondrial morphology between SMCs treated with different glucose concentrations in both CM and CaP conditions were observed (Figure 1F).

Next, we assessed whether increased ECM calcification attributed to glucose was due to a chemical or physical interaction between glucose and CaP. First, the nucleation of the CaP complexes was assessed in vitro within a cell‐free system. As expected, CM exhibited a significantly lower calcium concentration than CaP, while the various glucose and CaP conditions showed similar calcium levels (Figure S1A). Second, live and fixated/dead (previously fixed with 4% PFA) SMCs were cultured for 7 days in CM with 5.5 mM glucose or CaP at 0 mM, 5.5 mM, and 25 mM glucose, or corresponding mannitol concentrations. Alizarin Red staining revealed no ECM calcification in PFA‐fixated cells (Figure S1B). Third, the T50 assay, which measures the time for primary calcium phosphate particles (CPPs) to transform into secondary CPPs, showed that varying glucose concentrations (0 mM, 5.5 mM, and 25 mM) did not alter the T50 times (Figure S1C). Collectively, these results suggest that glucose does not alter CPP formation and that the effect of glucose on SMC calcification depends on cellular integrity.

### 3.2 Transcriptome and Metabolome Data Integration Revealed an Alteration of the Hypotaurine/Taurine Pathway in Hyperglycemia‐Induced Vascular Smooth Muscle Cell Calcification

Classic osteochondrogenic and SMC markers were analyzed at the gene expression level to investigate molecular mechanisms underlying the concentration‐dependent effect of glucose in SMC calcification. Glucose did not significantly alter mRNA expression of the osteogenic markers ALPL, RUNX2, BMP2, and ENPP1, the chondrogenic markers MSX2, SOX9, and the SMC marker TAGLN in CaP‐treated SMCs (Figure S2A–G). These findings suggest that the classical osteogenic/chondrogenic signaling pathways might play a minor role in hyperglycemia‐induced SMC calcification at these early time points.

Therefore, we employed an untargeted metabolomics and transcriptomics approach to identify novel molecular drivers for the described hyperglycemia‐induced SMC calcification.

**FIGURE 2:**
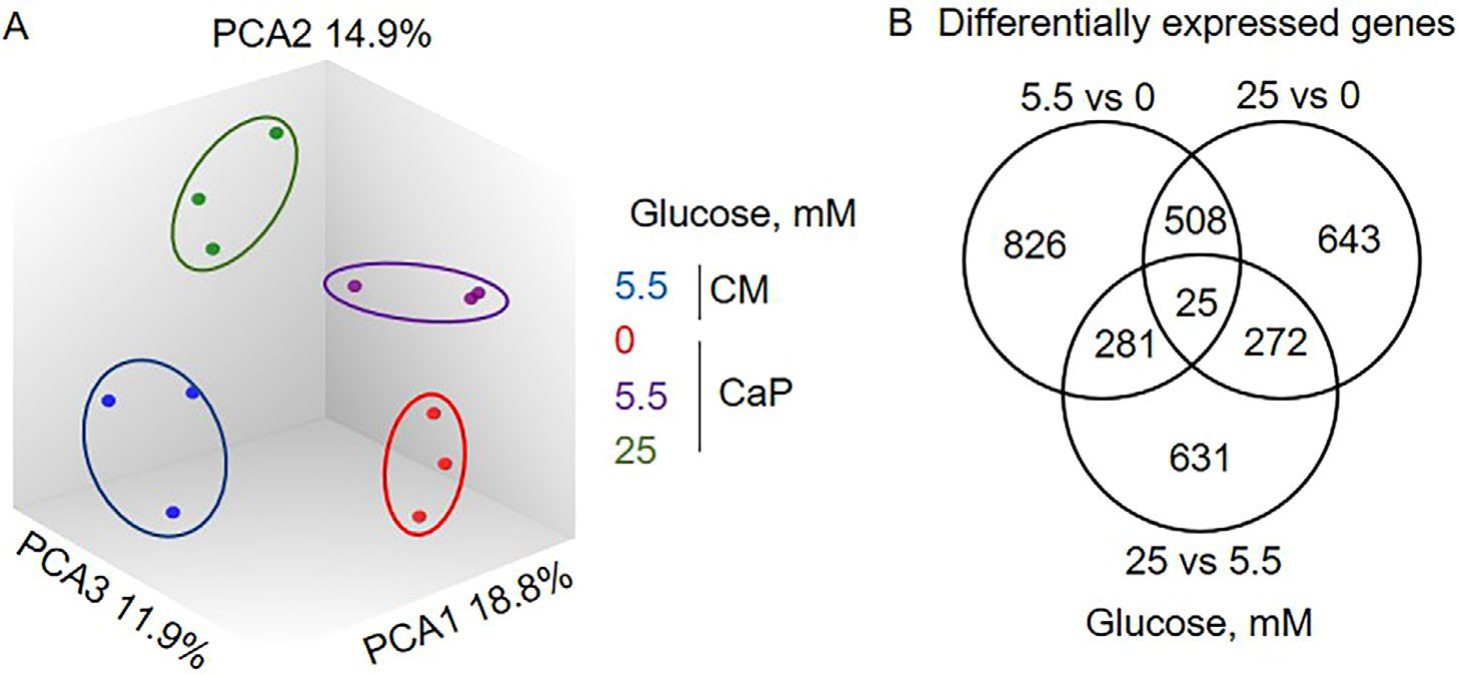
Distinct glucose concentration‐specific gene expression profiles in primary human coronary artery smooth muscle cells. (A) Principal component analysis plot showed the segregation of the genes according to control (CM; 5.5 mM glucose), calcium/phosphate (CaP), and glucose treatment. The percentages indicate the proportion of variance explained by each feature. (B) The Venn diagram displayed the shared and unique differentially expressed genes (fold change ±1.2, *p* < 0.05) between different glucose treatments in CaP. *n* = 3, each *n* represents an independent pSMC donor.

Transcriptomic analysis was performed on day 3, while metabolomics of both cells and supernatant was assessed on days 3 and 5 (Figure S3). These time points were chosen based on the observation that hyperglycemia‐induced ECM calcification became detectable by Alizarin Red staining by day 3 and was fully established by day 7. To investigate the mechanisms initiating calcification, we focused on an early time point (day 3) when calcification begins and a later one (day 5) when it is more advanced but not yet fully established. The same primary SMC donors were used for both transcriptomics and metabolomics. Principal component analysis (PCA) of the genes revealed distinct glucose concentration‐specific clustering (Figure 2A). Furthermore, there was an apparent clustering between CM and CaP. Differential gene expression analysis identified unique drivers of glucose‐mediated ECM calcification, with only 25 out of 3186 genes being shared between the glucose concentrations (fold change ±1.2, *p* < 0.05, Figure 2B and Table S2). To further determine the functional effect of glucose in calcifying SMCs, differentially expressed genes from the two extreme glucose concentrations (0 and 25 mM) were evaluated for pathway overrepresentation analysis. Pathways associated with cholesterol biosynthesis, activation of gene expression/cholesterol biosynthesis by sterol regulatory element‐binding proteins, and metabolism of steroids/lipids were at the top of the overrepresented pathways (Table S3). Notably, regarding the classic osteochondrogenic and SMC markers, ALPL, RUNX2, and ENPP1 were not significantly regulated when comparing 0 mM and 25 mM glucose.

**FIGURE 3:**
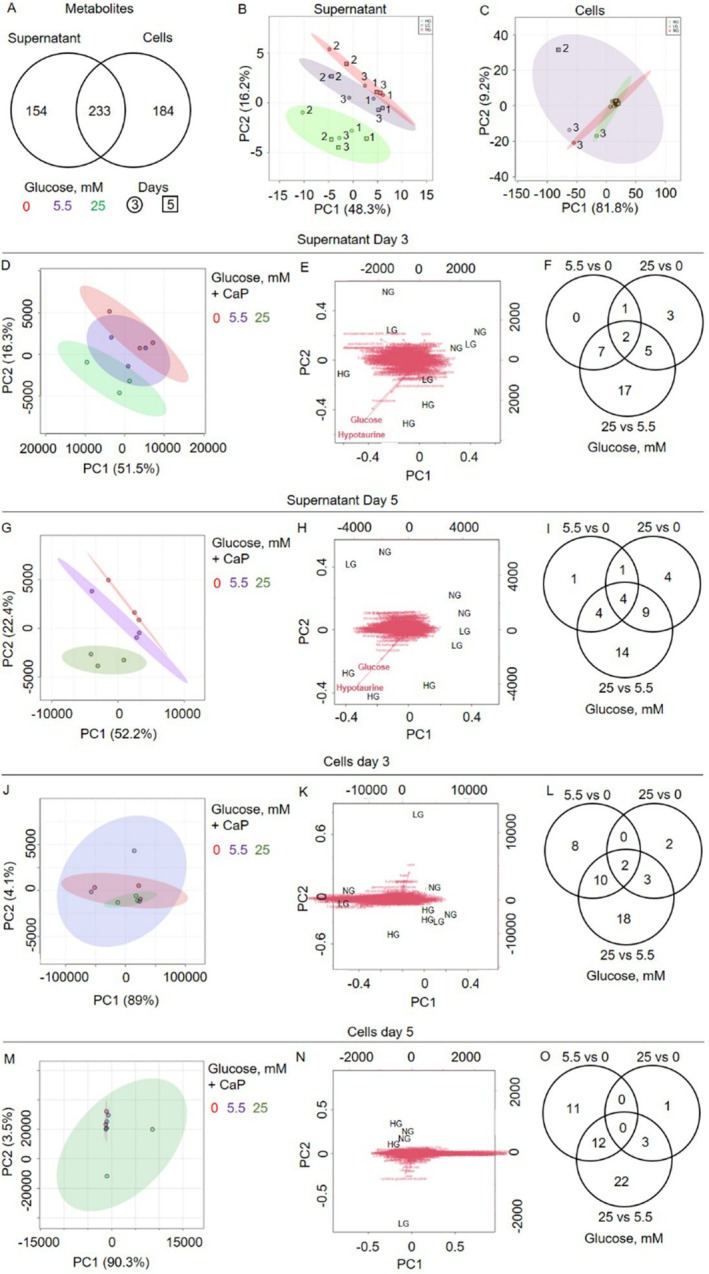
Glucose concentrations and time points shape unique metabolomic profiles in calcifying primary human coronary artery smooth muscle cells (pSMC). (A) Venn diagram displayed the shared and unique metabolites between supernatant and cells (calcifying SMCs). The principal component analysis (PCA) plot showed the segregation of the metabolites according to glucose treatment in (B) supernatant and (C) cells. PCA analysis was performed to distinguish between calcifying supernatant and cell samples across different timepoints. PCA biplots illustrate the positions of metabolites, with vectors indicating their relative contributions to the principal components. (D–F) Supernatant at day 3. (G–I) Supernatant at day 5. (J–L) Cell metabolites at day 3. (M–O) Cell metabolites at day 5. *n* = 3, each *n* represents an independent pSMC donor. Fold change ±1.2, *p* < 0.05.

Metabolomics analysis identified a total of 571 unique metabolites. Among these, SMCs revealed 184 exclusive intracellular metabolites, while 154 were solely detected in the supernatant (Figure 3A, Table S4). Cells and supernatants shared 40.8% of the metabolites (233 out of 571). A PCA scatter plot demonstrated that, in the supernatant, different glucose concentration groups formed distinct metabolite clusters (Figure 3B). In contrast, this distinction based on glucose was not evident for intracellular metabolites (Figure 3C).

Regarding the different time points, on day 3, metabolites in the supernatant formed 3 clusters that overlapped but were clearly separated on day 5, according to the PCA analysis (Figure 3D,G). Additionally, in the PCA biplot, the relative positions of metabolites indicate their relationships and contributions to the principal components. For days 3 and 5, glucose and hypotaurine, represented through vectors in the biplot, stand out due to their distance from other metabolites (Figure 3E,H). The observed role of glucose is consistent with expectations, as the cells were treated with glucose, resulting in measurable differences in glucose concentrations in the supernatant. However, the role of hypotaurine compared to other metabolites is a novel finding. In total, 35 metabolites were differentially abundant in the supernatant on day 3, and only two (glucose and fructosyllysine) were shared among all glucose concentrations (Figure 3F, Table S5A). At day 5, 37 metabolites were differentially abundant in the supernatant, with only four detected across all glucose concentration conditions (glucose, hypotaurine, 2‐oxo arginine, and fructosyllysine; Figure 3I, Table S5B).

The PCA analysis from the cell lysates did not show a clustering of samples related to glucose concentrations at both time points (Figure 3J,M). Moreover, the PCA biplot revealed that no metabolite significantly contributed to the principal components (Figure 3K,N). At day 3, 33 metabolites were differentially abundant in cells, and two metabolites (glycerol 3‐phosphate and N‐acetylglutamine) were shared among all glucose concentrations (Figure 3L, Table S5C). At day 5, 49 metabolites were differentially abundant in cells, and none of them were shared among all glucose concentrations (Figure 3O, Table S5D). The top overrepresented pathways from the 0 mM vs. 25 mM glucose comparison of the supernatant and cell metabolites at days 3 and 5 are listed in Tables S6–S9.

Next, aiming to identify metabolits associated with the cellular phenotype (0 mM glucose: no calcification, 25 mM glucose: increased calcification), a two‐way ANOVA analysis was performed, considering phenotype as one factor and time as the second factor. A total of 17 and 3 metabolites were significantly associated with the phenotype in the supernatant and cells, respectively (Figure S4A,C). The heatmaps display the abundance of those metabolites (Figure S4B,D). As a validation point, glucose in the supernatant was among the metabolites associated with the calcification phenotype (*p* < 0.001). Of note, 4‐(2‐hydroxyethyl)‐1‐piperazineethanesulfonic acid (HEPES, *p* = 9.95e‐5) was significantly associated with time in cells.

### 3.3 Transcriptome and Metabolome Data Integration Reveals the Hypotaurine/Taurine Pathway as a Potential Molecular Target in Hyperglycemia‐Induced Vascular Smooth Muscle Cell Calcification

**FIGURE 4:**
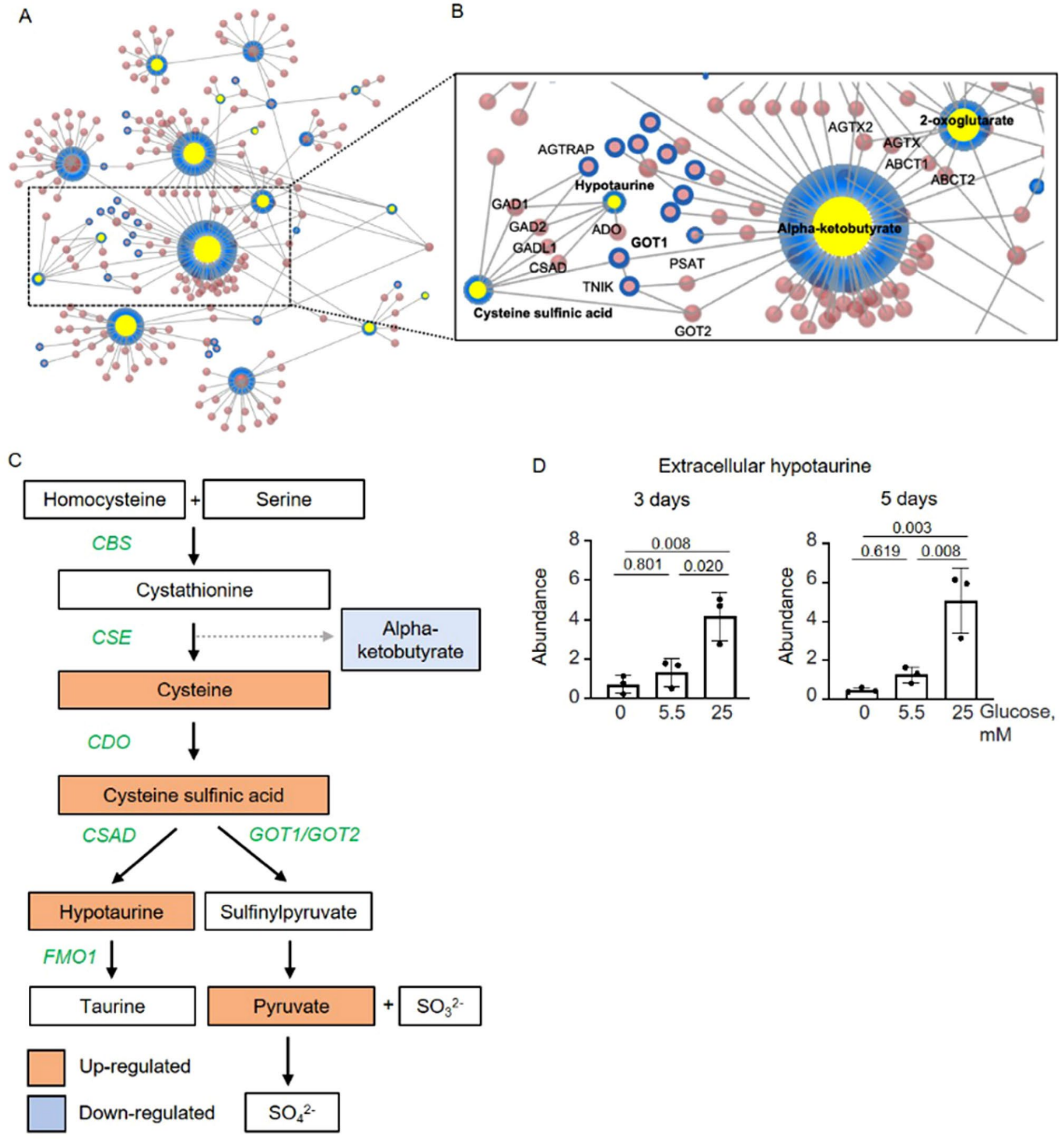
The multi‐omics network of hyperglycemia‐induced vascular calcification. (A) Multi‐omics network based on genes and metabolites differentially regulated, comparing 0 vs. 25 mM glucose treatment of calcifying human coronary artery smooth muscle cells. (B) Focused network on hypotaurine/taurine and cysteine metabolic pathways and their interactors. Blue nodes represent input molecules. Yellow nodes represent metabolites, and red nodes are based on protein–protein interactions from genes. Input: Differentially expressed genes (fold change 1.5, *p* < 0.05, day 3) and metabolites from the supernatant (fold change 1.2, *p* < 0.05, day 5) from the 0 vs. 25 mM glucose comparison. (C) Overview of the effect of glucose on the hypotaurine/taurine metabolic pathway. Blue: Decreased metabolites by glucose. Orange: Increased metabolites by glucose. White: Not regulated. Green: Enzyme gene names. (D) Abundance of secreted/extracellular hypotaurine based on an untargeted metabolomics approach. *n* = 3, each *n* represents an independent pSMC donor. Mean ± SD. One‐way ANOVA with Tukey's post hoc test.

To explore different mechanistic pathways from hyperglycemia‐induced ECM calcification, we integrated differentially expressed genes (fold change 1.2, *p* < 0.05, day 3) and metabolites from the supernatant (fold change 1.2, *p* < 0.05, day 5) from the comparison of 0 mM vs. 25 mM glucose to reconstruct a co‐expression network (Figure 4A). The interaction network identified center hub molecules related to the hypotaurine/taurine metabolic pathway, including metabolites such as cysteine, cysteine sulfinic acid, hypotaurine, alpha‐butyrate, as well as genes: acid cysteine sulfinic acid decarboxylase (CSAD) and aspartate aminotransferase, cytoplasmic (GOT1) (Figure 4B,C and Figure S5A–E).

Hypotaurine was secreted in a concentration‐dependent manner by glucose on both days 3 and 5 (Figure 4D), while its intracellular abundance remained unchanged based on the metabolomics quantified by mass spectrometry (Figure S5E). Interestingly, glucose treatment did not alter intracellular and secreted taurine—the product of hypotaurine metabolism and the final metabolite of the pathway (Figure S5F–G). Furthermore, intracellular lactate levels on day 3, as well as extracellular lactate levels on both days 3 and 5, were lower in the absence of glucose, indicating a reduction in glycolytic flux (Figure S6A,B). Additionally, extracellular pyruvate levels were higher in the presence of 25 mM glucose compared to 0 mM glucose on both days 3 and 5 (Figure S6D).

Therefore, our omics integration of metabolomics from the supernatant with transcriptomics suggests a role of the hypotaurine pathway in hyperglycemia‐induced SMC calcification.

### 3.4 Hypotaurine Reduces Extracellular Matrix Calcification of Vascular Smooth Muscle Cells

**FIGURE 5:**
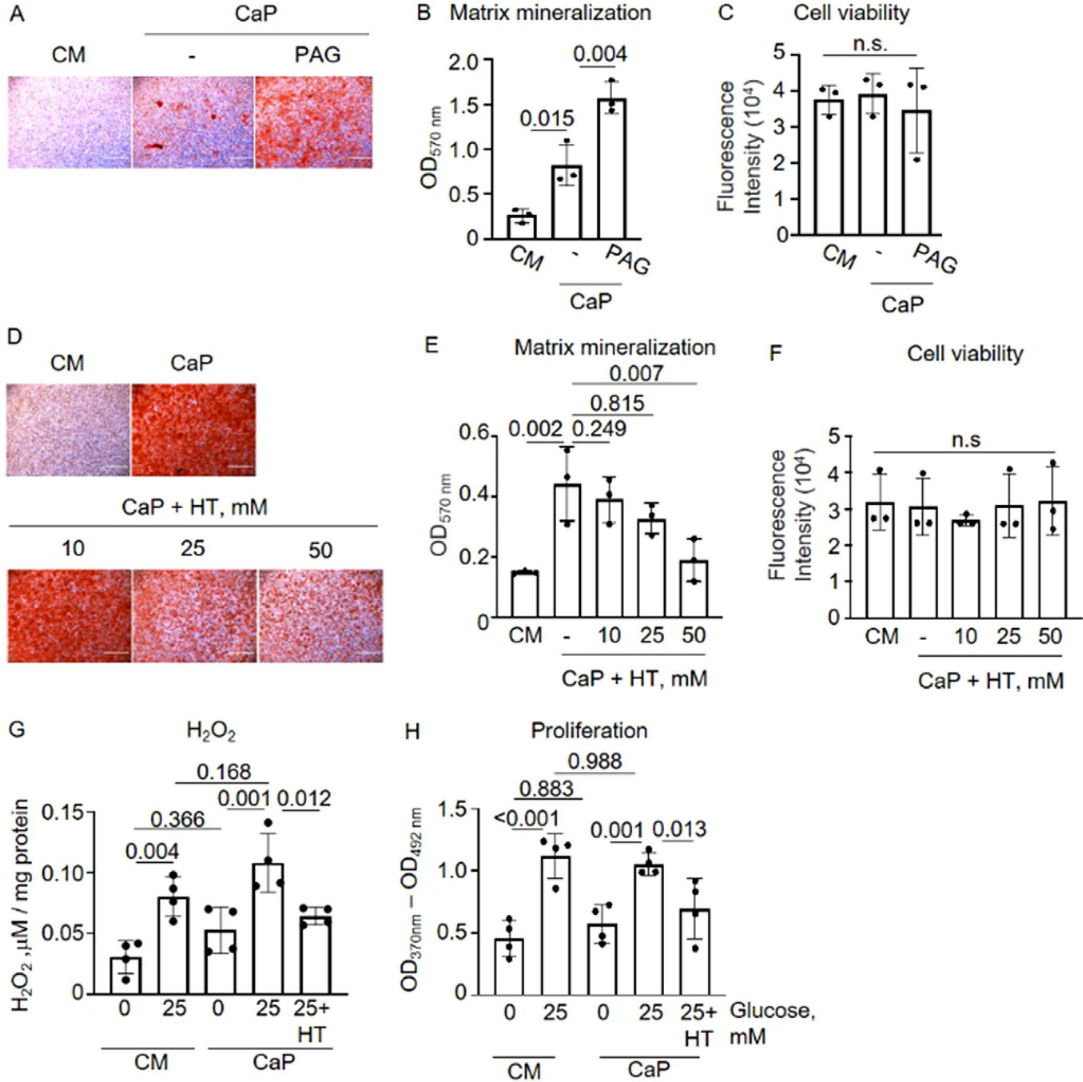
Hypotaurine reduced extracellular matrix (ECM) calcification in calcifying hyperglycemic immortalized vascular smooth muscle cells (imSMC), in a concentration‐ and time‐dependent manner. (A–C) imSMC were cultured in control (CM; 25 mM glucose) or calcium/phospate (CaP; 25 mM glucose) conditions with or without 1 μM PAG for 7 days. (A) Representative image of Alizarin Red S staining visualizing ECM mineral. Scale bar: 1000 μm. (B) Quantification of B by the elution of Alizarin Red stain. One‐way ANOVA with Sidak post hoc test. (C) Cell viability was assessed by alamarBlue assay. (D–F) imSMC were cultured in 25 mM glucose under control (CM) or calcium/phosphate (CaP) conditions without or with hypotaurine (10, 25, 50 mM). (D) Representative image of Alizarin Red S staining visualized ECM mineral. Day 7. Scale bar: 1000 μm. (E) Quantification of A by the elution of Alizarin Red stain. (F) Cell viability was accessed by AlamarBlue at day 7. Mean ± SD. (G, H) imSMC were cultured in 0 or 25 mM glucose under control (CM) or calcium/phosphate (CaP) conditions without or with 25 mM hypotaurine. (G) Oxidative stress was assessed by the Amplex Red hydrogen/peroxidase (H_2_O_2_) assay after 24 h and normalized to protein. (H) Cell proliferation was assessed by BrdU assay after 7 days. B‐F) *N* = 3, in duplicates. One‐way ANOVA with Dunnett's post hoc test compared to CaP, n.s.; not significant. G, H). *n* = 4, in duplicates. One‐way ANOVA with Sidak's post hoc test. Mean ± SD. Each *n* represents an independent experiment.

Next, we investigated the functional role of hypotaurine in SMC calcification. Propargyl glycine (PAG), an inhibitor of the upstream enzyme of hypotaurine–cystathionine gamma‐lyase cysteine (CSE, Figure 4C), was used to target hypotaurine production [28]. PAG treatment in the presence of 25 mM glucose and CaP increased ECM calcification 2‐fold (*p* = 0.004) compared to CaP alone (Figure 5A,B) without affecting cell viability (Figure 5C). Furthermore, hypotaurine treatment reduced ECM calcification in calcifying hyperglycemic SMCs concentration‐dependently, without affecting cell viability (Figure 5D–F) or CPP formation (Figure S7). To further validate these findings, we employed an independent SMC source—human ventral smooth muscle cells—and applied a novel fluorescent probe‐based technique that enables real‐time visualization of ECM mineralization. This complementary approach supported the dose‐dependent protective effect of hypotaurine against calcification under hyperglycemic conditions (Figure S8). These findings suggest a preventive compensatory role for hypotaurine secretion in SMC calcification.

Given the known antioxidant properties of hypotaurine [29], we next assessed oxidative stress levels by measuring H_2_O_2_ production in SMCs. We observed that glucose triggered a concentration‐dependent increase in H_2_O_2_ production, which was independent of the presence of CaP (Figure 5G). In hyperglycemic conditions with CaP treatment, hypotaurine mitigated H_2_O_2_ production, achieving comparable levels to 0 mM glucose.

Following the same pattern, 0 mM glucose reduced SMC proliferation in control (*p* = 0.001) and CaP conditions (*p* = 0.001) compared to 25 mM glucose (Figure 5H). Hypotaurine significantly reduced cell proliferation in calcifying hyperglycemic SMCs (*p* = 0.013).

### 3.5 Hypotaurine Restores the Disrupted Energy Metabolism in Calcifying Hyperglycemic Vascular Smooth Muscle Cells

**FIGURE 6:**
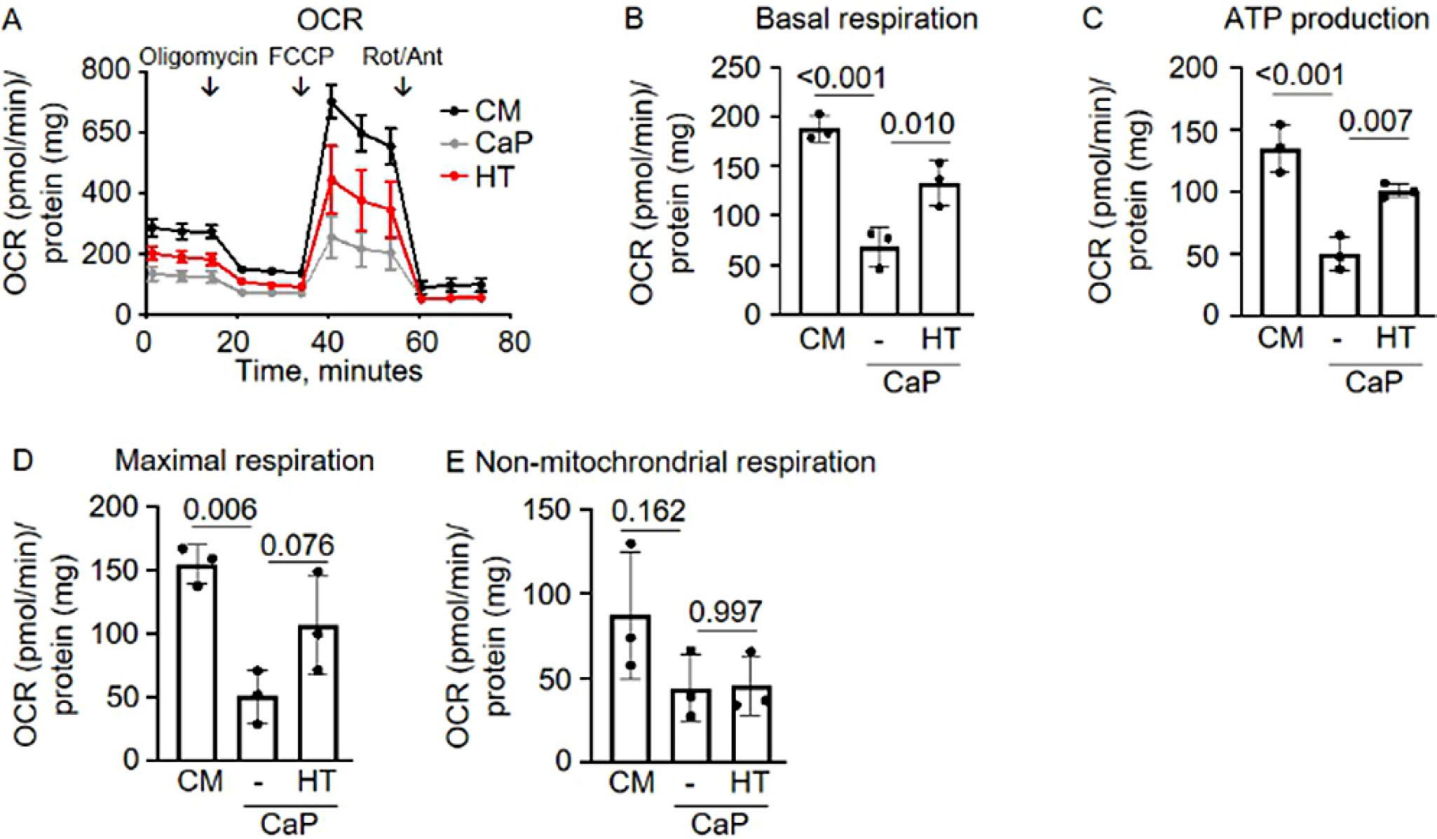
Hypotaurine partially restores mitochondrial respiration in calcifying hyperglycemic immortalized vascular smooth muscle cells (imSMC). imSMCs were cultured in 25 mM glucose for 7 days in either control medium (CM) or calcium phosphate (CaP) conditions with or without 25 mM hypotaurine. (A) Mitochondrial oxygen consumption rate (OCR) was measured using the Seahorse XF96 flux analyzer. Mitochondrial effectors were sequentially injected: oligomycin (ATP synthase inhibitor), FCCP (uncoupling agent), and rotenone/antimycin (complex I/III inhibitors), as indicated by downward arrows. (B) Basal respiration quantified after mitochondrial effector injections. (C) ATP production derived from changes in OCR after oligomycin injection. (D) Maximal respiration measured after FCCP injection, indicating the maximal electron transport chain capacity. (E) Non‐mitochondrial respiration calculated following rotenone/antimycin injections, representing OCR from non‐mitochondrial sources. All data were normalized to the protein content. Mean ± SD. *n* = 3, in duplicates. Each *n* represents an independent experiment. One‐way ANOVA with Sidak's post hoc test.

Mitochondria play a significant role in generating cellular ROS during oxidative phosphorylation, which drives ATP production. In previous work, we demonstrated that CaP‐mineralized SMCs treated with 25 mM glucose exhibit reduced mitochondrial respiration, as evidenced by real‐time extracellular efflux analysis [19]. Building upon this, we investigated whether hypotaurine treatment could reverse these adverse effects on mitochondrial bioenergetics. As anticipated, under 25 mM glucose conditions, CaP treatment decreased key mitochondrial phosphorylation parameters, including basal respiration, ATP production, and maximal respiration (Figure 6A–D). Interestingly, hypotaurine partially rescued all these adverse effects. Neither CaP nor hypotaurine altered non‐mitochondrial respiration (Figure 5E).

### 3.6 Sodium‐ and Chloride‐Dependent (Hypo)taurine Transporters Affect the Hyperglycemia‐Induced Extracellular Matrix Mineralization of Vascular Smooth Muscle Cells

Both taurine and hypotaurine are transported across the plasma membrane via the taurine/hypotaurine transporter SLC6A6 (TAUT protein), facilitating their entry into the intracellular space [30]. We observed an increase in TAUT protein expression under the 0 mM glucose calcification condition compared to the control (Figure S9A,B). Interestingly, while CaP treatment did not significantly upregulate TAUT expression in 25 mM glucose conditions, we noted a reduction in TAUT protein levels in these cells compared to those calcifying in 0 mM glucose. Specifically, SMCs calcifying in the presence of 25 mM glucose displayed lower levels of SLC6A6 protein than those calcifying under 0 mM glucose conditions. Furthermore, silencing SLC6A6 in calcifying SMCs promoted ECM calcification (Figure S9C–E), supporting the concept that SLC6A6 and the subsequent intracellular transport of hypotaurine play a role in mitigating calcification.

**FIGURE 7:**
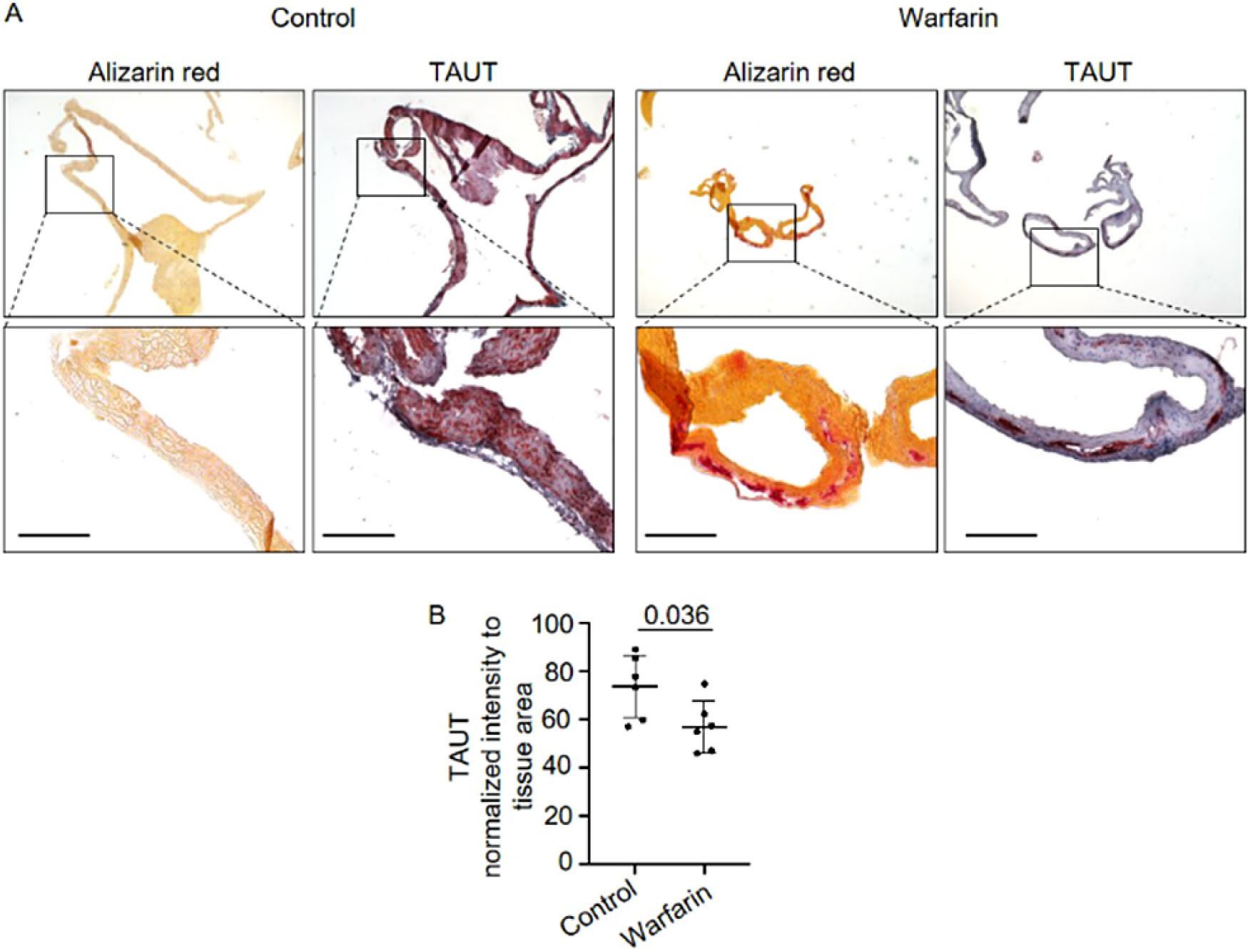
Sodium‐ and chloride‐dependent (hypo)taurine transporter SLC6A6 (protein TAUT) expression pattern in aortas with medial calcification from warfarin‐treated mice. (A) Representative images of control (*n* = 6) and warfarin‐treated (*n* = 6) mice. Calcification is visualized by Alizarin red staining. Immunohistochemistry was performed for TAUT. Bar: 150 μm. (B) Quantification of positively stained areas for TAUT in murine aortas using ImageJ v2.0. Mean ± SD. Each dot depicts one mouse. Student's *t*‐test.

Finally, to validate our in vitro findings, we employed a murine warfarin‐induced model of VC and examined TAUT expression in the aortic arch using immunohistochemistry and histological analysis. Calcified regions were identified by Alizarin Red staining. In the warfarin‐treated group, inhibition of vitamin K‐dependent proteins led to increased calcium deposition in vascular tissues, particularly within SMCs (Figure 7A). In contrast to control animals, which exhibited strong TAUT staining in SMCs, warfarin‐treated mice showed a marked reduction in TAUT expression (Figure 7A,B).

Together, these results suggest that SLC6A6/TAUT may play a protective role in mitigating VC.

## 4 Discussion

Our results demonstrated a concentration‐dependent stimulation of ECM calcification by glucose, with an unexpected finding that the absence of glucose fully inhibited ECM calcification. Notably, although glucose was absent in the basal DMEM medium, SMCs were cultured in 10% FBS containing glucose, resulting in a final concentration of approximately 0.37 mM. Importantly, our results showed that only living cells calcify the ECM, supporting the notion that glucose‐induced calcification is a cell‐mediated biological process rather than a physical–chemical reaction between glucose and CaP. While previous research has linked cell death with calcification, suggesting apoptotic cells act as nucleation sites for mineral deposition, our findings indicated that glucose‐induced calcification occurred independently of cell death [31, 32]. This was further supported by the observation that 25 mM glucose significantly increased calcification without affecting SMC viability.

Consistent with the literature, high‐glucose concentrations exacerbate oxidative stress, promoting cytotoxicity through increased H_2_O_2_ production [33, 34]. However, while glucose did not induce cell death in our study, it enhanced H_2_O_2_ levels, likely contributing to calcification. Calcification of SMCs is typically associated with a phenotypic shift toward an osteoblast‐like state [35], characterized by expression of osteogenic markers such as ALPL [36, 37], OPN [36], osteocalcin [36], SOX9 [36, 37], and BMP2 [38], alongside a reduction in SMC markers like α‐SMA [39]. Contrary to this classical view, our study revealed no significant alterations in these osteogenic or chondrogenic pathways, suggesting that hyperglycemia‐induced calcification may involve non‐chondro‐osteogenic mechanisms, at least up to the 7‐day culture period employed. We performed untargeted transcriptomic analysis to explore the underlying pathways, revealing that glucose profoundly affected gene expression. Only 0.8% of the differentially expressed genes overlapped between the different glucose concentrations, and PCA showed distinct gene clusters corresponding to glucose levels. Interestingly, while metabolic changes are expected during VC, few studies have explored the metabolomic landscape of VC. Notably, only a single metabolomics study related to VC was found in the literature in which metabolomics was performed in blood samples of patients with intracranial and extracranial carotid atherosclerosis [40].

Our metabolomic analysis of hyperglycemia‐induced in vitro SMC calcification revealed that extracellular metabolites, rather than intracellular ones, were most strongly associated with glucose concentration. While much of the VC research to date has focused on intracellular signaling pathways and biomarkers, our findings underscore the importance of the extracellular milieu in calcification. Integrating transcriptomics and metabolomics data, we reconstructed a hyperglycemia‐induced multi‐omics network, identifying the hypotaurine/taurine metabolic pathway as a central hub.

Taurine, the most abundant free amino acid in humans, plays key roles in osmotic pressure regulation, neuromodulation, intracellular calcium modulation, and antioxidant defense [41, 42]. Clinical trials have demonstrated taurine's potential to reduce hypertension and hypercholesterolemia, improve oxidative stress, and reduce inflammatory markers in diabetic patients [43, 44, 45]. Young type 1 diabetic patients given oral taurine supplementation for two weeks displayed reversal of both arterial stiffness and brachial artery reactivity [44]. In Japan, taurine is approved for treating congestive heart failure [46]. In an atherosclerosis mouse model, taurine was shown to reduce LDL oxidation and protect endothelial cells from oxidative stress and apoptosis and reduce adhesion molecules like ICAM‐1, further supporting its protective cardiovascular role [47, 48]. Interestingly, our data show that taurine levels did not change during hyperglycemia‐induced in vitro SMC calcification. Despite these benefits, no studies have yet examined hypotaurine in the context of diabetes or VC. Unlike taurine, hypotaurine cannot be obtained from the diet; it is synthesized exclusively through the decarboxylation of cysteine sulfinic acid [49]. Although its biological role remains unclear, hypotaurine is thought to act as an osmolyte, a cell cycle regulator, and a potent antioxidant [29].

Our study identified hypotaurine as a significantly regulated metabolite in response to glucose. Blockage of the hypotaurine/taurine pathway enhanced ECM calcification, while supplementation with hypotaurine reduced calcification, suggesting a protective role in preventing hyperglycemia‐induced in vitro SMC calcification. Mechanistically, hypotaurine reduced H_2_O_2_ levels in calcifying SMCs treated with high glucose, implying that its protective effect may be linked to attenuating oxidative stress—a key driver of VSMC calcification.

Beyond the role of oxidative stress and hypotaurine in hyperglycemia‐induced SMC in vitro calcification, our findings suggest that lipid metabolism is also affected, potentially contributing to SMC dysfunction and ECM mineralization. Besides glucose, SMCs can also use fatty acids as their energy source [50]. Chronic hyperglycemia disrupts lipid metabolism, altering cholesterol biosynthesis and steroid/lipid pathways, which may contribute to lipid accumulation in SMCs, a key feature of vascular dysfunction in diabetes [51]. Our transcriptomic analysis revealed dysregulated fatty acid and cholesterol metabolism in calcifying SMCs under different glucose conditions. Notably, beta‐oxidation molecules, particularly carnitine‐conjugated fatty acids, were among the most affected metabolites, suggesting a metabolic shift in SMCs. Given the high energy demands of the SMC phenotypic switch, fatty acid oxidation may serve as an adaptive energy source. Supporting this, glucose depletion in ex vivo rabbit aorta reduced contractile function, which was partially restored by fatty acids like butyrate and oleate [52]. Interestingly, silencing carnitine O‐octanoyltransferase, a key enzyme in fatty acid metabolism, increased ECM mineralization, further implicating metabolic remodeling in SMC calcification [53]. These findings highlight the need to investigate alternative metabolic pathways driving VC in hyperglycemia. Despite this metabolic flexibility, the link between fatty acid metabolism and VC remains to be further explored.

The role of mitochondria in VC is increasingly recognized, particularly in the context of disrupted energy metabolism and oxidative phosphorylation [54]. Our previous findings demonstrated impaired mitochondrial phosphorylation in CaP‐calcified SMCs [19], and interestingly, we observed that hypotaurine partially restored these mitochondrial functions. This suggests that the protective effect of hypotaurine may extend beyond its role in antioxidant defense, including modulation of mitochondrial metabolism.

Finally, our findings regarding SLC6A6/TAUT expression and its relations to calcification provide additional mechanistic findings into the protective role of the hypotaurine/taurine pathway in VC. The observation that TAUT expression increased under glucose‐depleted calcification conditions while decreasing in high‐glucose conditions, suggests a potential adaptive response mechanism. Our in vivo findings demonstrating reduced TAUT expression in murine arteries of warfarin‐induced calcification align with a previous study reporting diminished TAUT expression in human atherosclerotic femoral arteries and rat carotid arteries undergoing neointima formation [55]. This observation is particularly interesting given that SLC6A6 is abundantly expressed in SMCs [56] and functions as a regulator of SMC proliferation by inhibiting the Wnt/β‐catenin signaling pathway [55]. Importantly, SLC6A6 maintains the contractile phenotype of SMCs by upregulating the expression of contractile markers, including SM22α and α‐SMA [55], suggesting that its downregulation during calcification may represent a transition point where SMCs become more susceptible to phenotypic modulation and ECM mineralization. Our experimental data demonstrating enhanced ECM mineralization following SLC6A6 silencing further supports the protective role of this transport system, likely mediated through its ability to facilitate the cellular uptake of both taurine and hypotaurine.

In conclusion, our findings offer new insights into glucose‐induced SMC metabolic pathways—specifically those involving hypotaurine—and that targeting this pathway may protect against VC, particularly in T2DM patients. This study also paves the way for future investigations into non‐classical pathways and extracellular metabolites in calcification processes, providing a broader understanding of VC in complex metabolic environments.

## Author Contributions

**Marina A. Heuschkel:** conceptualization, investigation, writing – original draft, visualization. **Armand Jaminon:** investigation. **Steffen Gräber:** investigation. **Anna Artati:** investigation, methodology. **Jerzy Adamski:** investigation, methodology. **Joachim Jankowski:** investigation. **Leon Schurgers:** writing – review and editing, resources, funding acquisition, supervision. **Nikolaus Marx:** writing – review and editing, resources. **Willi Jahnen‐Dechent:** writing – review and editing. **Claudia Goettsch:** conceptualization, funding acquisition, writing – review and editing, project administration, supervision, resources.

## Conflicts of Interest

Leon Schurgers receives grants from Gnosis by Lesaffre, Bayer, and Boehringer Ingelheim and is a shareholder of Coagulation Profile. All other authors declare no conflicts of interest.

## Supporting information


**Figure S1.** Glucose did not affect calcium/phosphate (CaP) complex building. (A) Calcium content was measured in control (CM; 5.5 mM glucose) and CaP media with varying glucose (0 mM, 5.5 mM, and 25 mM) and its corresponding mannitol concentrations after 7 days of incubation in the absence of cells. Mean ± SD. *N* = 3. One‐way ANOVA with Sidak’s post hoc test. (B) Representative images of extracellular matrix mineral using Alizarin Red staining for live and 4% paraformaldehyde‐fixed primary human coronary artery smooth muscle cells treated with glucose and mannitol in CaP for 7 days. Scale bars: 1000 μm. *N* = 3. (C) T50 values in response to different glucose concentrations. Based on a turbidity test, the T50 assay measures the half‐maximum transformation time of primary calciproteins into secondary calciproteins. Glucose was diluted in 140 mM NaCl solution to a final concentration of 0, 5, 25, and 25 mM. Data are presented as the mean of two independent experiments in duplicate.
**Figure S2.** Calcium/phosphate (CaP) treatment and glucose did not alter the mRNA expression of the classic osteogenic, chondrogenic, and smooth muscle cell markers. Primary human coronary artery smooth muscle cells (pSMC) were cultured in control (CM; 5.5 mM glucose) or CaP‐enriched media with 0, 5.5, or 25 mM glucose for 7 days. mRNA levels were quantified by qPCR. (A) Tissue‐nonspecific alkaline phosphatase (ALPL). (B) Runt‐related transcription factor (RUNX2). (C) Bone morphogenetic protein 2 (BMP2). (D) Ectonucleotide pyrophosphatase/phosphodiesterase family 1 (ENPP1). (E) Homeobox protein MSX2 (MSX2). (F) Transcription factor SOX9 (SOX9). (G) Transgelin (TAGLN). *N* = 3, each *n* represents an independent pSMC donor. Mean ± SD. One‐way ANOVA with Sidak’s post hoc test, n.s.; not significant.
**Figure S3.** Experimental design for the multi‐omics approach. Three independent primary human coronary artery smooth muscle cell donors were cultured for up to 5 days in control (CM; 5.5 mM glucose) and calcium/phosphate (CaP) medium with 0, 5.5, and 25 mM glucose. Transcriptomics was performed on day 3. Metabolomics from cells and supernatant were performed on days 3 and 5.
**Figure S4.** Two‐way ANOVA analysis evaluated the metabolomic changes associated with the phenotype (0 and 25 mM glucose) and time (3 and 5 days) in calcifying primary human coronary artery smooth muscle cells cultured in 0 or 25 mM glucose. Venn diagram for the number of metabolites that had a significant *p* value (< 0.05) regarding the phenotype, time, or the interaction of both factors for (A) supernatant and (C) cells. Heatmap of the abundance of the metabolites identified as significant for phenotype analysis in the (B) supernatant and (D) cells. Metabolites in the heatmap are sorted by clusters. Fold change ±1.2.
**Figure S5.** The abundance of metabolites from the hypotaurine/taurine pathway is based on an untargeted metabolomics approach of calcifying primary human coronary smooth muscle cells (pSMC). pSMC were cultured in calcium/phosphate (CaP)‐enriched media with 0, 5.5, or 25 mM glucose for 3 and 5 days. The abundance of (A) intracellular serine, (B) extracellular cysteine sulfinic acid, (C) extracellular alpha‐ketobutyrate, (D) extracellular pyruvate, (E) intracellular hypotaurine, (F) extracellular taurine, and (G) intracellular taurine according to time. *N* = 3, each *n* represents an independent pSMC cell donor. Mean ± SD. One‐way ANOVA with Tukey’s post hoc test, n.s.; not significant.
**Figure S6.** The abundance of intracellular and extracellular lactate is based on an untargeted metabolomics approach of calcifying primary human coronary smooth muscle cells (pSMC). pSMC were cultured in calcium/phosphate (CaP)‐enriched media with 0, 5.5, or 25 mM glucose for 3 and 5 days. The abundance of (A) intracellular lactate and (B) extracellular lactate. *N* = 3, each *n* represents an independent pSMC cell donor. Mean ± SD. One‐way ANOVA with Tukey’s post hoc test, n.s.; not significant.
**Figure S7.** Hypotaurine did not affect calcium/phosphate (CaP) complex building based on the T50 turbidity test. Hypotaurine was diluted in 140 mM NaCl solution to a final concentration of 5, 25, and 50 mM. Data are presented as the mean of two independent experiments in duplicate.
**Figure S8.** Hypotaurine reduced ECM calcification in calcifying hyperglycemic human ventral smooth muscle cells. Cells were cultured in 25 mM glucose under calcium/phosphate (CaP) media with or without hypotaurine (10, 25, 50 mM). Mineralization was visualized using live‐time fluorescence imaging using Alexa Fluor‐546‐tagged fetuin‐A (orange) as a calcification sensor merged to phase contrast (gray/black). Representative images from *n* = 3 independent human ventral smooth muscle cell donors (in duplicates). Scale bar: 1000 μm.
**Figure S9.** Silencing of SLC6A6 promoted extracellular matrix (ECM) calcification in calcifying hyperglycemic immortalized vascular smooth muscle cells (imSMC). Human vascular immortalized smooth muscle cells (imSMCs) were cultured in 0 mM or 25 mM glucose under control (CM) and calcium/phosphate (CaP) conditions for 7 days. (A) Protein expression of the taurine/hypotaurine receptor (TAUT; *SLC6A6*) was accessed by Western blot and (B) quantified. (C) *SLC6A6* was silenced, and the mRNA expression was quantified by qPCR. (D) The alizarin red staining visualized ECM mineralization, and (E) it eluted for quantification. Mean ± SD. Fold increase to 0 mM glucose CM. *n* = 3–4 in duplicates; each *n* represents an independent experiment. One‐way ANOVA with Sidak’s post hoc test.

**Table S1.** TaqMan probes used for real‐time PCR.
**Table S2.** Differentially expressed genes according to transcriptomics analysis in primary human coronary artery smooth muscle cells treated with different glucose concentrations for 3 days. Fold change ±1.2, *p* < 0.05. *Excel file*.
**Table S3.** Pathway overrepresentation analysis of the differentially expressed genes between 0 vs. 25 mM glucose treatment in calcified human coronary artery smooth muscle cells (ConsensusPathDB).
**Table S4.** Metabolites identified in the cell and supernatant of primary human coronary artery smooth muscle cells according to metabolomics analysis. Fold change ±1.2, *p* < 0.05.
*Excel file*.
**Table S5.** Differentially expressed metabolites according to metabolomics analysis in primary human coronary artery smooth muscle cells and supernatant treated with different glucose concentrations for 3 or 5 days. Fold change ±1.2, *p* < 0.05.
*Excel file*.
**Table S6.** Pathway overrepresentation analysis of differentially expressed metabolites (fold change ±1.2, *p* < 0.001) between 0 and. 25 mM glucose in the supernatant of calcified cells at day 3 (MetaboAnalyst).
**Table S7.** Pathway overrepresentation analysis of differentially expressed metabolites (fold change ±1.2, *p* < 0.001) between 0 and 25 mM glucose in the supernatant of calcified cells at day 5 (MetaboAnalyst).
**Table S8.** Pathway overrepresentation analysis of differentially expressed metabolites (fold change ±1.2, *p* < 0.001) between 0 and 25 mM glucose in calcified cells at day 3 (MetaboAnalyst).
**Table S9.** Pathway overrepresentation analysis of differentially expressed metabolites (fold change ±1.2, *p* < 0.001) between 0 and 25 mM glucose in calcified cells at day 5 (MetaboAnalyst).

## Data Availability

The data that support the findings of this study are available from the corresponding author upon reasonable request.
