# Supplementary material for: Hypotaurine Reduces Glucose‐Mediated Vascular Calcification
Source: Acta Physiol (Oxf). 2025 Jul 7;241(8):e70075. doi: 10.1111/apha.70075 (PMC12230643; doi:10.1111/apha.70075)
Supplement: Supplementary file 1 — Figure S1. Glucose did not affect calcium/phosphate (CaP) complex building. (A) Calcium content was measured in control (CM; 5.5 mM glucose) and CaP media with varying glucose (0 mM, 5.5 mM, and 25 mM) and its corresponding mannitol concentrations after 7 days of incubation in the absence of cells. Mean ± SD. N = 3. One‐way ANOVA with Sidak’s post hoc test. (B) Representative images of extracellular matrix mineral using Alizarin Red staining for live and 4% paraformaldehyde‐fixed primary human coronary artery smooth muscle cells treated with glucose and mannitol in CaP for 7 days. Scale bars: 1000 μm. N = 3. (C) T50 values in response to different glucose concentrations. Based on a turbidity test, the T50 assay measures the half‐maximum transformation time of primary calciproteins into secondary calciproteins. Glucose was diluted in 140 mM NaCl solution to a final concentration of 0, 5, 25, and 25 mM. Data are presented as the mean of two independent experiments in duplicate. Figure S2. Calcium/phosphate (CaP) treatment and glucose did not alter the mRNA expression of the classic osteogenic, chondrogenic, and smooth muscle cell markers. Primary human coronary artery smooth muscle cells (pSMC) were cultured in control (CM; 5.5 mM glucose) or CaP‐enriched media with 0, 5.5, or 25 mM glucose for 7 days. mRNA levels were quantified by qPCR. (A) Tissue‐nonspecific alkaline phosphatase (ALPL). (B) Runt‐related transcription factor (RUNX2). (C) Bone morphogenetic protein 2 (BMP2). (D) Ectonucleotide pyrophosphatase/phosphodiesterase family 1 (ENPP1). (E) Homeobox protein MSX2 (MSX2). (F) Transcription factor SOX9 (SOX9). (G) Transgelin (TAGLN). N = 3, each n represents an independent pSMC donor. Mean ± SD. One‐way ANOVA with Sidak’s post hoc test, n.s.; not significant. Figure S3. Experimental design for the multi‐omics approach. Three independent primary human coronary artery smooth muscle cell donors were cultured for up to 5 days in control (CM; 5.5 mM glucose) and calc [file APHA-241-e70075-s002.docx]

**Hypotaurine reduces glucose-mediated vascular calcification**

**Supplementary figures**

**Supplementary Figure 1**

**
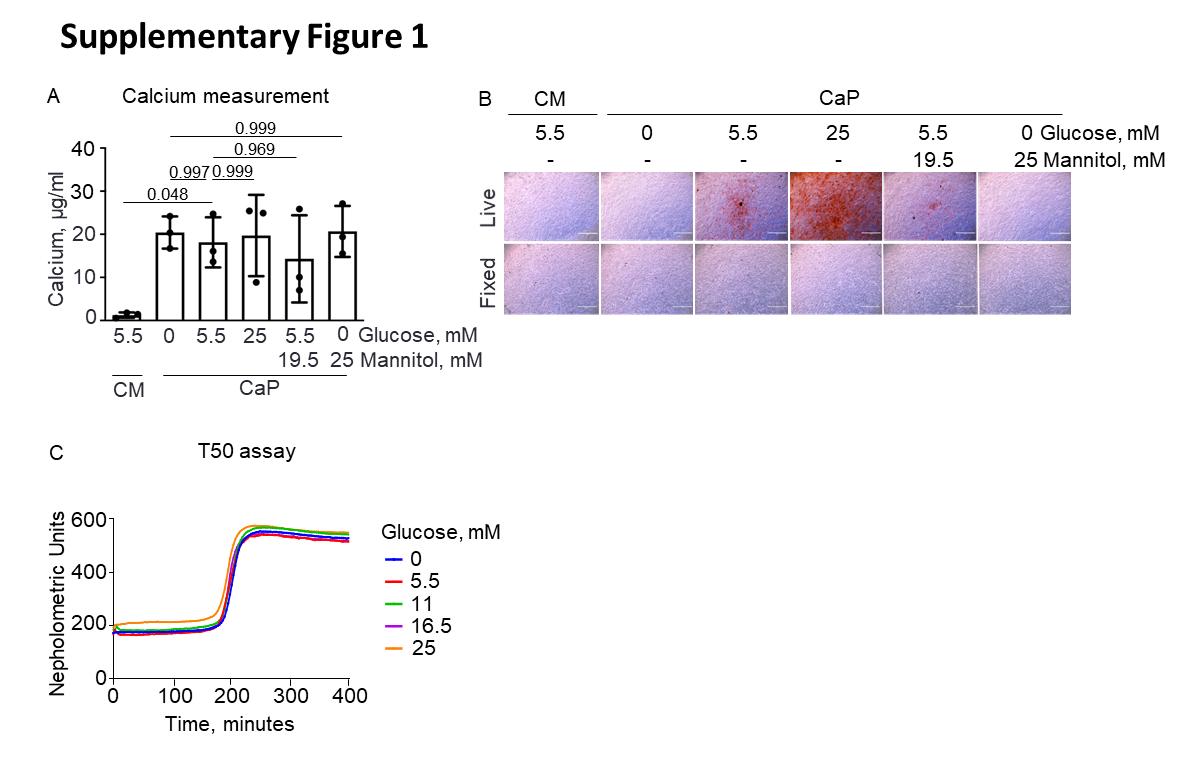
**

**Supplementary Figure 1.** **Glucose did not affect calcium/phosphate (CaP) complex building. A)** Calcium content was measured in control (CM; 5.5 mM glucose) and CaP media with varying glucose (0 mM, 5.5 mM and 25 mM) and its corresponding mannitol concentrations after 7 days of incubation in the absence of cells. Mean ± SD. N = 3. One-way ANOVA with Sidak’s post hoc test. **B)** Representative images of extracellular matrix mineral using Alizarin Red staining for live and 4 % paraformaldehyde-fixed primary human coronary artery smooth muscle cells treated with glucose and mannitol in CaP for 7 days. Scale bars: 1000 µm. N = 3. **C)** T50 values in response to different glucose concentrations. Based on a turbidity test, T50 assay measures the half-maximum transformation time of primary calciproteins into secondary calciproteins. Glucose was diluted in 140 mM NaCl solution to a final concentration of 0, 5, 25 and 25 mM. Data are presented as mean of two independent experiments in duplicates.

**Supplementary Figure 2**


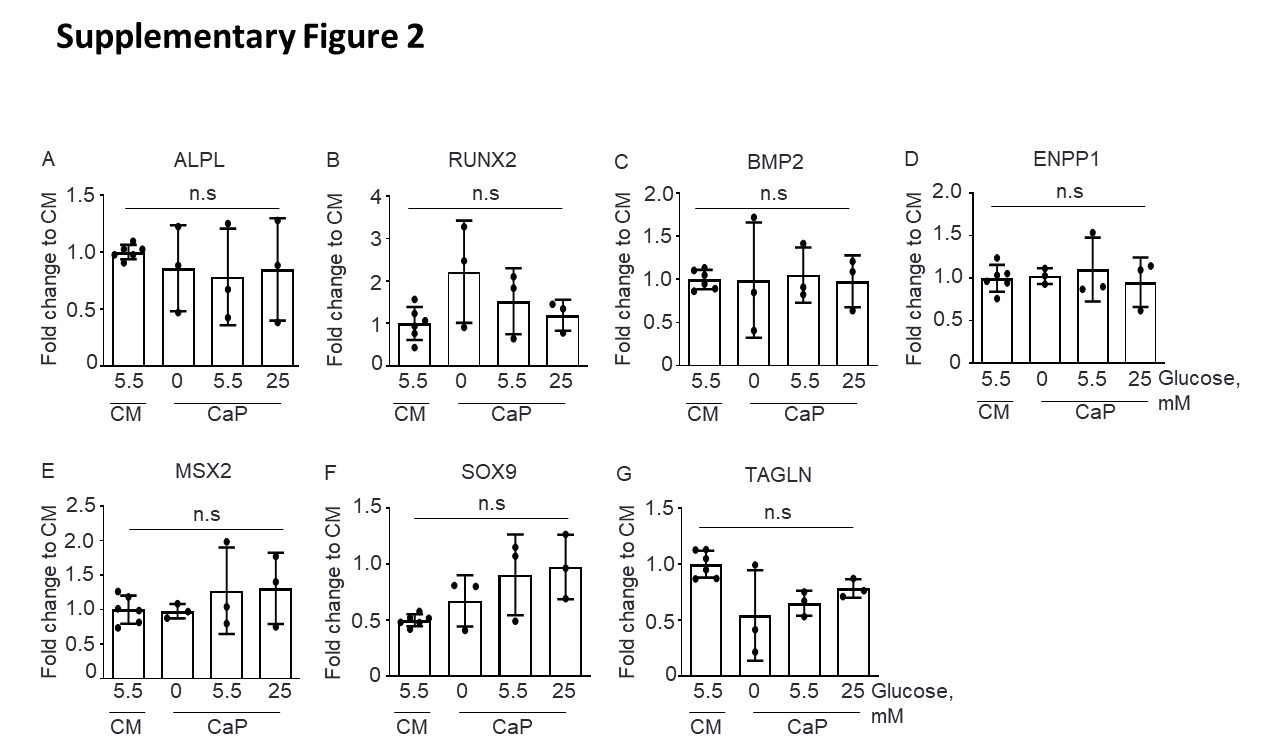


**Supplementary Figure 2.** **Calcium/phosphate (CaP) treatment and glucose did not alter the mRNA expression of the classic osteogenic, chondrogenic, and smooth muscle cell markers.** Primary human coronary artery smooth muscle cells (pSMC) were cultured in control (CM; 5.5 mM glucose) or CaP-enriched media with 0, 5.5, or 25 mM glucose for 7 days. mRNA levels were quantified by qPCR. **A)** tissue-nonspecific alkaline phosphatase (ALPL), **B)** Runt-related transcription factor (RUNX2), **C)** Bone morphogenetic protein 2 (BMP2), **D)** Ectonucleotide pyrophosphatase / phosphodiesterase family 1 (ENPP1), **E)** Homeobox protein MSX2 (MSX2), **F)** Transcription factor SOX9 (SOX9), and **G)** Transgelin (TAGLN). N = 3, each n represents an independent pSMC donor. Mean ± SD. One-way ANOVA with Sidak’s post hoc test, n.s; not significant.

**Supplementary Figure 3**


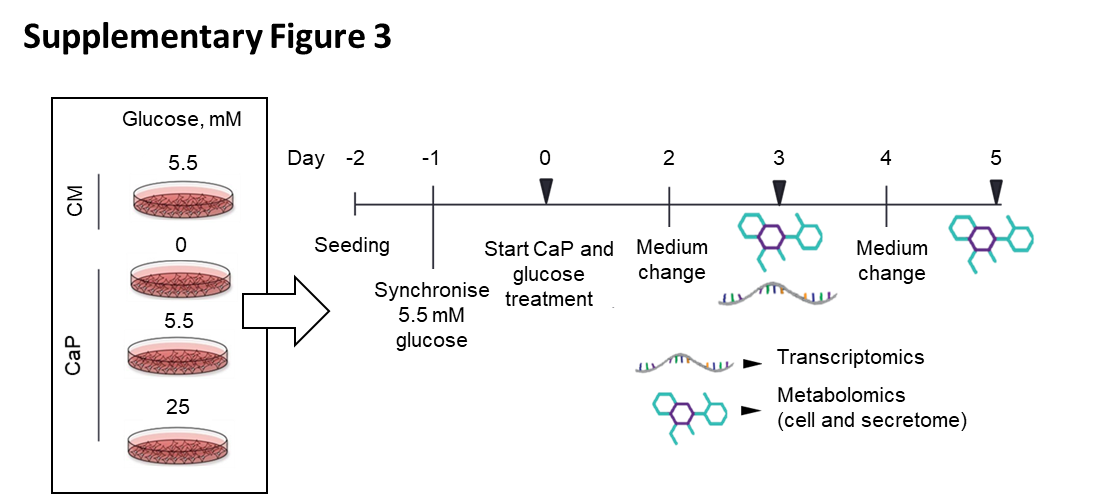


**Supplementary Figure 3.** **Experimental design for the multi-omics approach.** Three independent primary human coronary artery smooth muscle cell donors were cultured for up to 5 days in control (CM; 5.5 mM glucose) and calcium/phosphate (CaP) medium with 0, 5.5, and 25 mM glucose. Transcriptomics was performed on day 3. Metabolomics from cells and supernatant were performed on days 3 and 5.

**Supplementary Figure 4**


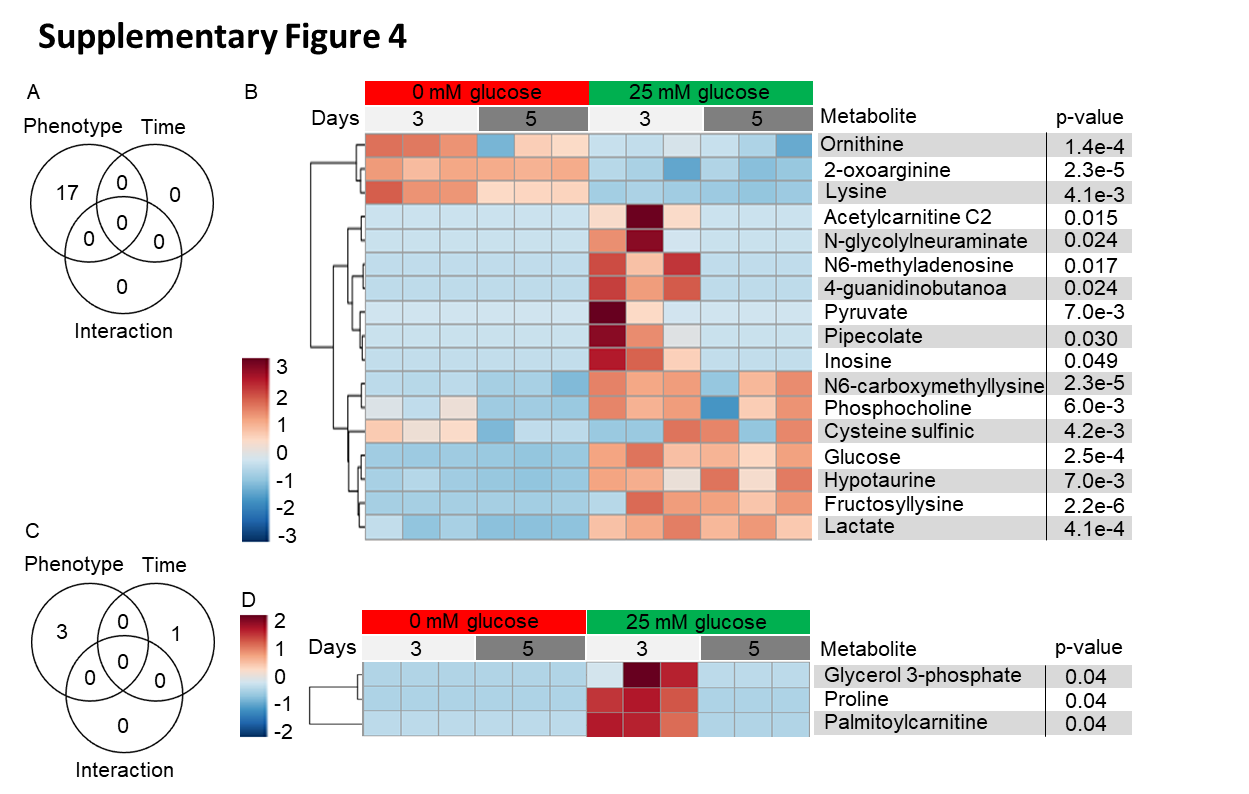


**Supplementary Figure 4. Two-way ANOVA analysis evaluated the metabolomic changes associated with the phenotype (0 and 25 mM glucose) and time (3 and 5 days) in calcifying primary human coronary artery smooth muscle cells cultured in 0 or 25 mM glucose.** Venn diagram for the number of metabolites that had a significant p-value (< 0.05) regarding the phenotype, time, or the interaction of both factors for **A)** supernatant and **C)** cells. Heatmap of the abundance of the metabolites identified as significant for phenotype analysis in the **B)** supernatant and **D)** cells. Metabolites in the heatmap are sorted by clusters. Fold change ±1.2.

**Supplementary Figure 5**

**
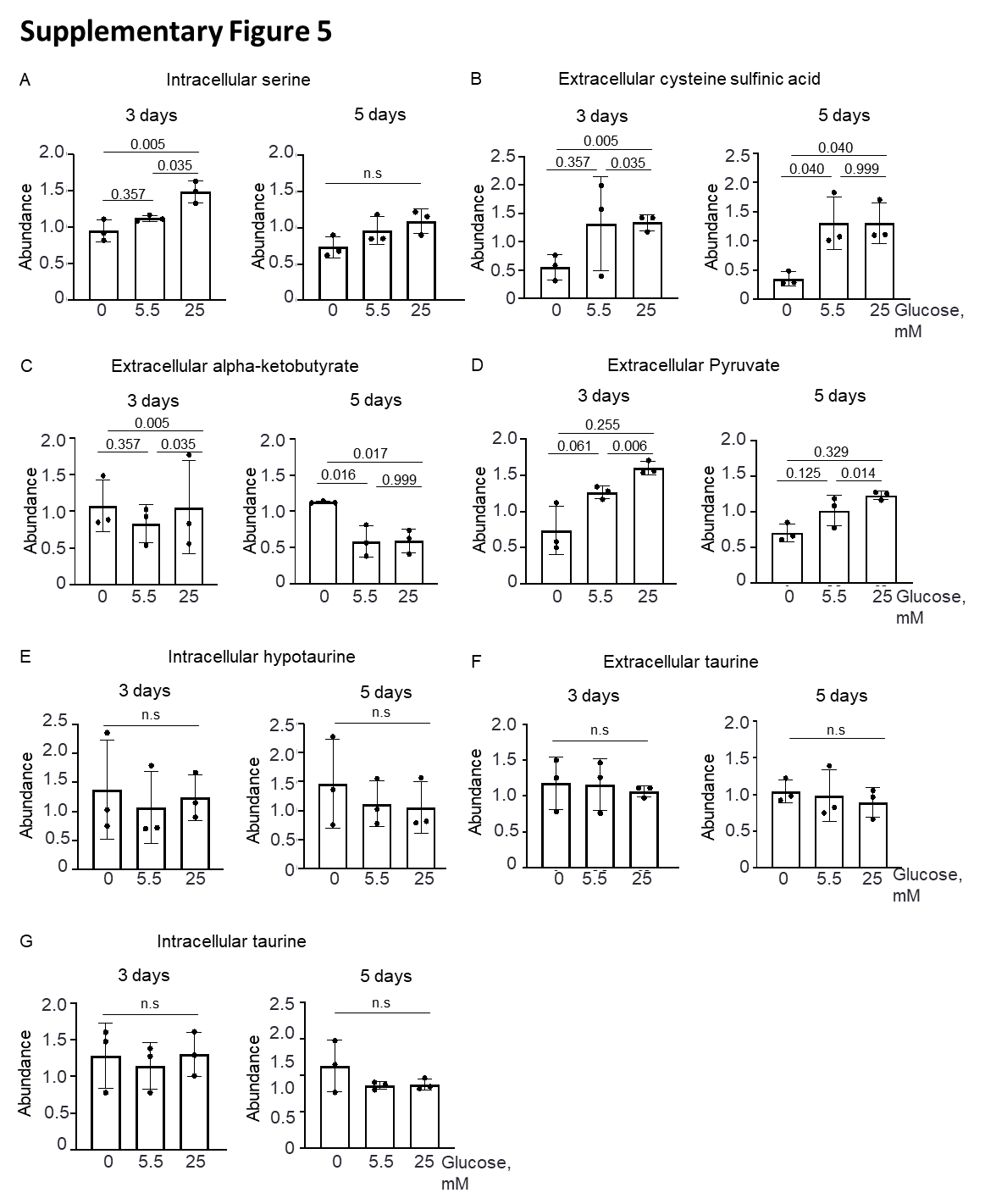
**

**Supplementary Figure 5. The abundance of metabolites from the hypotaurine/taurine pathway based on an untargeted metabolomics approach of calcifying primary human coronary smooth muscle cells (pSMC).** pSMC were cultured in calcium/phosphate (CaP)-enriched media with 0, 5.5, or 25 mM glucose for 3 and 5 days. The abundance of **A)** intracellular serine, **B)** extracellular cysteine sulfinic acid, **C)** extracellular alpha-ketobutyrate, **D)** extracellular pyruvate, **E)** intracellular hypotaurine, **F)** extracellular taurine, and **G)** intracellular taurine according to time. N = 3, each n represents an independent pSMC cell donor. Mean ± SD. One-way ANOVA with Turkey’s post hoc test, n.s; not significant.

**Supplementary Figure 6**


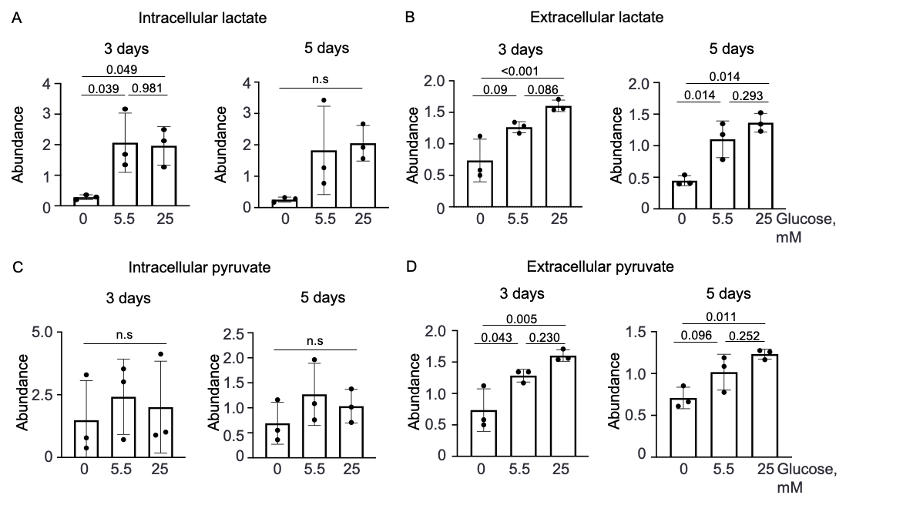


**Supplementary Figure 6. The abundance of intracellular and extracellular lactate based on an untargeted metabolomics approach of calcifying primary human coronary smooth muscle cells (pSMC).** pSMC were cultured in calcium/phosphate (CaP)-enriched media with 0, 5.5, or 25 mM glucose for 3 and 5 days. The abundance of **A)** intracellular lactate, **B)** extracellular lactate. N = 3, each n represents an independent pSMC cell donor. Mean ± SD. One-way ANOVA with Turkey’s post hoc test, n.s; not significant.

**Supplementary Figure 7**


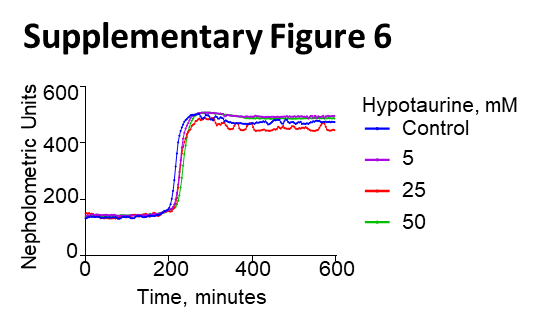


**Supplementary Figure 7.** **Hypotaurine did not affect calcium/phosphate (CaP) complex building based on T50 turbidity test.** Hypotaurine was diluted in 140 mM NaCl solution to a final concentration of 5, 25 and 50 mM. Data are presented as mean of two independent experiments in duplicates.

**Supplementary Figure 8**


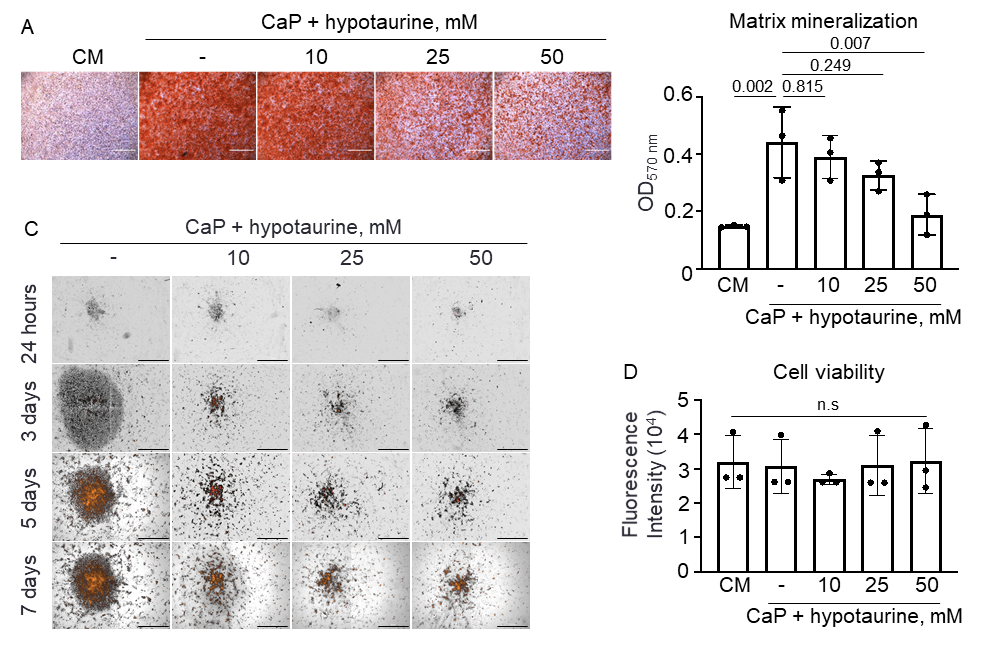


**Supplementary Figure 8.** **Hypotaurine reduced ECM calcification in calcifying hyperglycemic human ventral smooth muscle cells.** Cells were cultured in 25 mM glucose under calcium/phosphate (CaP) media with or without hypotaurine (10, 25, 50 mM). Mineralization was visualized using live-time fluorescence imaging using Alexa Fluor®-546-tagged fetuin-A (orange) as a calcification sensor merged to phase contrast (gray/black). Representative images from n = 3 independent human ventral smooth muscle cell donors (in duplicates). Scale bar: 1000 μm.

**Supplementary Figure 9**


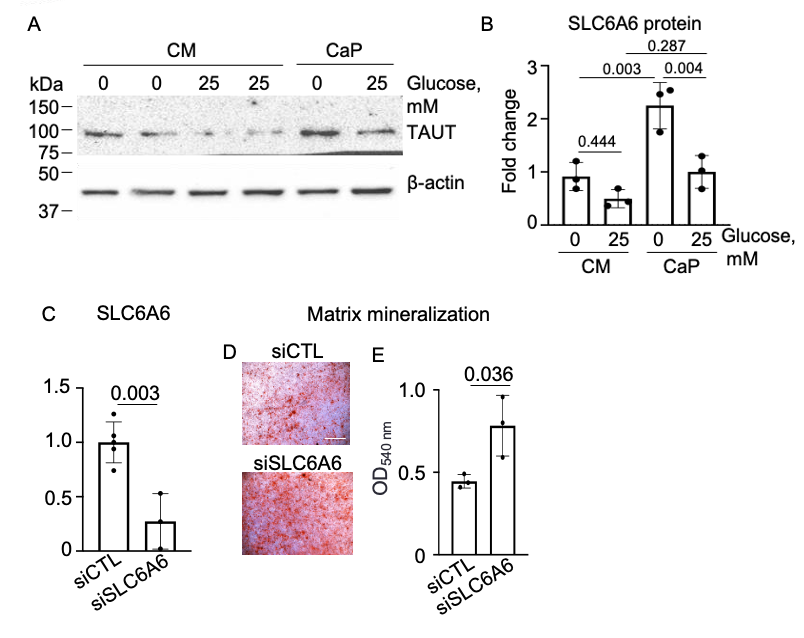

**Supplementary Figure 9.** **Silencing of SLC6A6 promoted extracellular matrix calcification in calcifying hyperglycemic immortalized vascular smooth muscle cells (imSMC).** Human vascular immortalized smooth muscle cells (imSMCs) were cultured in 0 mM or 25 mM glucose under control (CM) and calcium/phosphate (CaP) conditions for 7 days. A) Protein expression of the taurine/hypotaurine receptor (TAUT; *SLC6A6*) was accessed by western blot and B) quantified. C) *SLC6A6* was silenced, and the mRNA expression was quantified by qPCR. D) The alizarin red staining visualized extracellular matrix mineralization and E) eluted for quantification. Mean ± SD. Fold increase to 0 mM glucose CM. n = 3-4 in duplicates, each n represents an independent experiment. One-way ANOVA with Sidak’s post hoc test.
