# Supplementary material for: Hypotaurine Reduces Glucose‐Mediated Vascular Calcification
Source: Acta Physiol (Oxf). 2025 Jul 7;241(8):e70075. doi: 10.1111/apha.70075 (PMC12230643; doi:10.1111/apha.70075)
Supplement: Supplementary file 2 — Table S1. TaqMan probes used for real‐time PCR. Table S2. Differentially expressed genes according to transcriptomics analysis in primary human coronary artery smooth muscle cells treated with different glucose concentrations for 3 days. Fold change ±1.2, p < 0.05. Excel file. Table S3. Pathway overrepresentation analysis of the differentially expressed genes between 0 vs. 25 mM glucose treatment in calcified human coronary artery smooth muscle cells (ConsensusPathDB). Table S4. Metabolites identified in the cell and supernatant of primary human coronary artery smooth muscle cells according to metabolomics analysis. Fold change ±1.2, p < 0.05. Excel file. Table S5. Differentially expressed metabolites according to metabolomics analysis in primary human coronary artery smooth muscle cells and supernatant treated with different glucose concentrations for 3 or 5 days. Fold change ±1.2, p < 0.05. Excel file. Table S6. Pathway overrepresentation analysis of differentially expressed metabolites (fold change ±1.2, p < 0.001) between 0 and. 25 mM glucose in the supernatant of calcified cells at day 3 (MetaboAnalyst). Table S7. Pathway overrepresentation analysis of differentially expressed metabolites (fold change ±1.2, p < 0.001) between 0 and 25 mM glucose in the supernatant of calcified cells at day 5 (MetaboAnalyst). Table S8. Pathway overrepresentation analysis of differentially expressed metabolites (fold change ±1.2, p < 0.001) between 0 and 25 mM glucose in calcified cells at day 3 (MetaboAnalyst). Table S9. Pathway overrepresentation analysis of differentially expressed metabolites (fold change ±1.2, p < 0.001) between 0 and 25 mM glucose in calcified cells at day 5 (MetaboAnalyst). [file APHA-241-e70075-s001.zip › Heuschkel et al. _Supplementary Tables 1, 3, 6-9_APH-2025-01-0002R1.docx]

**Supplementary tables**

**Supplementary Table 1. TaqMan probes used for real-time PCR.**

| **Gene** | **Species** | **Probe** |
| --- | --- | --- |
| RPLP0 | Human | Hs99999902_m1 |
| RUNX2 | Human | Hs01047978_m1 |
| BMP2 | Human | Hs00154192_m1 |
| ENPP1 | Human | Hs01054040_m1 |
| MSX2 | Human | Hs00741177_m1 |
| SOX9 | Human | Hs00165814-m1 |
| TAGLN | Human | Hs01038777-g1 |

**Supplementary Table 3.** Pathway overrepresentation analysis of the differentially expressed genes between 0 vs 25 mM glucose treatment in calcified human coronary artery smooth muscle cells (ConsensusPathDB).

| **Pathway name** | **Candidates contained** | **p-value** | **q-value** | **Pathway source** |
| --- | --- | --- | --- | --- |
| Cholesterol biosynthesis | 16/25 (64.0%) | 5.9e-20 | 3.7e-17 | Reactome |
| Activation of gene expression by SREBF | 13/26 (50.0%) | 1.7e-14 | 5.3e-12 | Reactome |
| Metabolism of steroids | 23/127 (18.1%) | 1.9e-13 | 4.1e-11 | Reactome |
| Regulation of cholesterol biosynthesis by SREBP | 13/31 (41.9%) | 3.0e-13 | 4.7e-11 | Reactome |
| Steroid biosynthesis | 10/19 (52.6%) | 1.0e-11 | 1.2e-09 | KEGG |
| Metabolism of lipids | 46/664 (7.0%) | 1.0e-09 | 1.1e-07 | Reactome |
| Metabolism | 88/1972 (4.5%) | 1.3e-07 | 1.1e-05 | Reactome |
| Metallothioneins bind metals | 6/12 (50.0%) | 2.5e-07 | 1.9e-05 | Reactome |
| Cholesterol biosynthesis via desmosterol | 4/4 (100.0%) | 4.6e-07 | 2.9e-05 | Reactome |
| Cholesterol biosynthesis via lathosterol | 4/4 (100.0%) | 4.6e-07 | 2.9e-05 | Reactome |
| Response to metal ions | 6/15 (40.0%) | 1.2e-06 | 7.3e-05 | Reactome |
| Lysosome | 14/123 (11.4%) | 4.1e-06 | 2.1e-4 | KEGG |
| Terpenoid backbone biosynthesis | 6/22 (27.3%) | 1.6e-05 | 7.8e-4 | KEGG |

**Supplementary Table 6.** Pathway overrepresentation analysis of differentially expressed metabolites (fold change ±1.2, p-value < 0.001) between 0 vs 25 mM glucose in the supernatant of calcified cells at day 3 (MetaboAnalyst).

| **Pathway name** | **Candidates contained** | **p-value** | **q-value** |
| --- | --- | --- | --- |
| Arginine and proline metabolism | 5/36 (9.0%) | 9.06e-6 | 7.25e-4 |

**Supplementary Table 7.** Pathway overrepresentation analysis of differentially expressed metabolites (fold change ±1.2, p-value < 0.001) between 0 vs 25 mM glucose in the supernatant of calcified cells at day 5 (MetaboAnalyst).

| **Pathway name** | **Candidates contained** | **p-value** | **q-value** |
| --- | --- | --- | --- |
| Glycerophospholipid metabolism | 4/36 (16.6%) | 2.8e-4 | 2.2e-2 |
| Glyoxylate and dicarboxylate metabolism | 3/31 (9.6%) | 2.8e-3 | 0.113 |
| Arginine biosynthesis | 2/14 (14.2%) | 7.5e-3 | 0.194 |
| Glycerolipid metabolism | 2/16 (12.5%) | 9.8e-3 | 0.194 |

**Supplementary Table 8.** Pathway overrepresentation analysis of differentially expressed metabolites (fold change ±1.2, p-value < 0.001) between 0 vs 25 mM glucose in calcified cells at day 3 (MetaboAnalyst).

| **Pathway name** | **Candidates contained** | **p-value** | **q-value** |
| --- | --- | --- | --- |
| Glycine, serine and threonine metabolism | 3/33 (9.0%) | 2.2e-3 | 0.115 |
| Glycerophospholipid metabolism | 3/36 (8.3%) | 2.8e-3 | 0.115 |
| Arginine biosynthesis | 2/14 (14.2%) | 5.6e-3 | 0.148 |
| Glycerolipid metabolism | 2/16 (12.5%) | 7.4e-3 | 0.148 |

**Supplementary Table 9.** Pathway overrepresentation analysis of differentially expressed metabolites (fold change ±1.2, p-value < 0.001) between 0 vs 25 mM glucose in calcified cells at day 5 (MetaboAnalyst).

| **Pathway name** | **Candidates contained** | **p-value** | **q-value** |
| --- | --- | --- | --- |
| Glucose-Alanine Cycle | 3/13 (23.0%) | 3.5e-4 | 1.97e-2 |
| Gluconeogenesis | 4/33 (12.1%) | 4.0e-3 | 1.97e-2 |
| Warbung effect | 4/57 (7.0%) | 3.3e-3 | 7.38e-2 |
| Urea Cycle | 3/28 (14.2%) | 3.6e-3 | 7.38e-2 |
| Glycine and Serine Metabolism | 4/59 (6.7%) | 3.7e-3 | 7.38e-2 |
